# Importance of extended protease substrate recognition motifs in steering BNIP-2 cleavage by human and mouse granzymes B

**DOI:** 10.1186/1471-2091-15-21

**Published:** 2014-09-10

**Authors:** Petra Van Damme, Kim Plasman, Giel Vandemoortele, Veronique Jonckheere, Sebastian Maurer-Stroh, Kris Gevaert

**Affiliations:** 1 Department of Medical Protein Research, VIB, Flanders Interuniversity Institute for Biotechnology, Ghent University, A. Baertsoenkaai 3, B9000 Ghent, Belgium; 2 Department of Biochemistry, Ghent University, B-9000 Ghent, Belgium; 3 Bioinformatics Institute (BII), Agency for Science, Technology and Research (A*STAR), Singapore 138671, Singapore; 4 School of Biological Sciences (SBS), Nanyang Technological University (NTU), Singapore 637551, Singapore

**Keywords:** BNIP-2, Bid, Granzyme B, Extended substrate specificity, N-terminal COFRADIC, Near-cognate translation initiation, Degradomics

## Abstract

**Background:**

Previous screening of the substrate repertoires and substrate specificity profiles of granzymes resulted in long substrate lists highly likely containing bystander substrates. Here, a recently developed degradomics technology that allows distinguishing efficiently from less efficiently cleaved substrates was applied to study the degradome of mouse granzyme B (mGrB).

**Results:**

*In vitro* kinetic degradome analysis resulted in the identification of 37 mGrB cleavage events, 9 of which could be assigned as efficiently targeted ones. Previously, cleavage at the IEAD_75_ tetrapeptide motif of Bid was shown to be efficiently and exclusively targeted by human granzyme B (hGrB) and thus not by mGrB. Strikingly, and despite holding an identical P4-P1 human Bid (hBid) cleavage motif, mGrB was shown to efficiently cleave the BCL2/adenovirus E1B 19 kDa protein-interacting protein 2 or BNIP-2 at IEAD_28_. Like Bid, BNIP-2 represents a pro-apoptotic Bcl-2 protein family member and a potential regulator of GrB induced cell death. Next, *in vitro* analyses demonstrated the increased efficiency of human and mouse BNIP-2 cleavage by mGrB as compared to hGrB indicative for differing Bid/BNIP-2 substrate traits beyond the P4-P1 IEAD cleavage motif influencing cleavage efficiency. Murinisation of differential primed site residues in hBNIP-2 revealed that, although all contributing, a single mutation at the P3′ position was found to significantly increase the mGrB/hGrB cleavage ratio, whereas mutating the P1′ position from I_29_ > T yielded a 4-fold increase in mGrB cleavage efficiency. Finally, mutagenesis analyses revealed the composite BNIP-2 precursor patterns to be the result of alternative translation initiation at near-cognate start sites within the 5′ leader sequence (5′UTR) of BNIP-2.

**Conclusions:**

Despite their high sequence similarity, and previously explained by their distinct tetrapeptide specificities observed, the substrate repertoires of mouse and human granzymes B only partially overlap. Here, we show that the substrate sequence context beyond the P4-P1 positions can influence orthologous granzyme B cleavage efficiencies to an unmatched extent. More specifically, in BNIP-2, the identical and hGrB optimal IEAD tetrapeptide substrate motif is targeted highly efficiently by mGrB, while this tetrapeptide motif is refractory towards mGrB cleavage in Bid.

## Background

While gene orthology implies homology between two genes separated by a speciation event, no conclusions regarding their full functional equivalence can be simply extrapolated. Contradictions in literature on apparent isofunctional orthologs are thus partly due to the fact that incorrect functional extrapolations between orthologous genes were made. Human granzyme B (hGrB), a protease belonging to a family of serine proteases present in the granules of cytotoxic lymphocytes (cytotoxic T lymphocytes and natural killer cells) and implicated in the induction of cell death, was shown to display distinct functional and structural characteristics as compared to its murine ortholog despite their 80% amino acid similarity and 70% amino acid identity [1-4]. Positional scanning synthetic combinatorial libraries, phage display data and proteome-wide degradome analyses all aided in the elucidation of the differential specificity profiles and substrate repertoires of these orthologous granzymes [1-5]. Next to the higher cytotoxicity of hGrB, differences in cleavage efficiencies of Bid and caspase-3, substrates known to be of critical importance for the execution of hGrB induced cell death, were found for the human and mouse orthologous granzymes B [2-4]. While hGrB is capable of cleaving Bid and caspase-3 orthologs with comparable cleavage efficiencies, mGrB is unable to process Bid and cleaves mouse caspase-3 about 12-fold more efficient as compared to human caspase-3 [4]. Differential positional proteomics analyses furthermore reported on a proteome-wide analysis of the extended specificity profiles (i.e., beyond cleavage positions P4-P1) of both orthologous granzymes, and showed enhanced mGrB versus hGrB cleavage efficiencies of substrates holding Lys and Gly residues in the P1′ and P2′ positions respectively (P*n*-…-P2-P1-P1′-P2′-…-P*n’* nomenclature according to Schechter and Berger [6]) amongst other (subtle) distinct substrate traits [1].

The ability to separate critical from bystander cleavage events may also aid in elucidating the different underlying mechanisms of action engaged by these orthologous granzymes when exerting their primary functions [7-9]. Despite hindering a direct extrapolation to the physiological importance of mGrB-induced cleavages, distinctive orthologous granzyme B substrate traits can be captured using identical proteome backgrounds. As such, and in line with a previously developed positional proteomics strategy to follow substrate cleavage kinetics [8], we here made use of a human proteome background for identifying mGrB cleavage events. In this pioneering study on hGrB, of the 101 hGrB cleavage sites identified, only 18 were found to be efficiently cleaved, including cleavage at IEAD_75_ in the BH3-interacting domain death agonist Bid [Swiss-Prot: P55957] and at IEAD_28_ in the pro-apoptotic Bcl-2 family member BNIP-2 [Swiss-Prot: Q12982] [10,11]. While Bid stands central in hGrB induced apoptosis [12], both human and mouse Bid were found to be very poor mGrB substrates [1-4]. The essential requirement of Bid cleavage for hGrB-induced cell death [13] and the Bid independent cytotoxicity of mGrB [14], led to the postulation that mGrB exerts its cytotoxicity via direct activation of an effector caspase (i.e., caspase 3 and/or 7) [2], by relieving IAP inhibition of caspase-3, as shown for hGrB [15], and/or by the direct cleavage of yet unidentified substrates.

**Figure 1:**
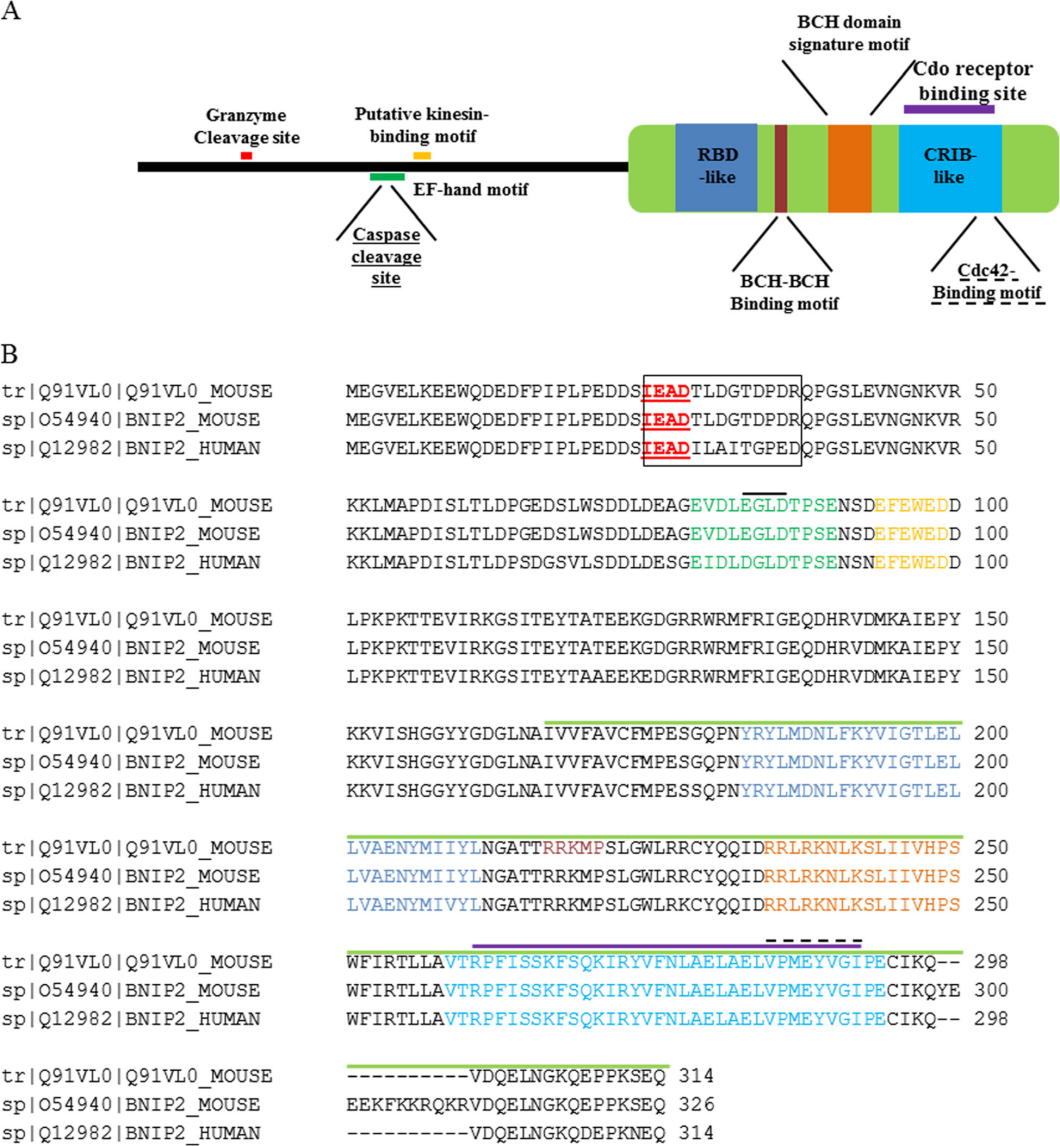
**Domain architecture and sequences of human and mouse BNIP-2. A**. Known or putative functional motifs indicated are: the Rho-binding domain (RBD), Cdc42/Rac1-Interactive Binding (CRIB)-like motif, BCH signature motif, BCH/BCH interaction motif, caspase and granzyme cleavage sites and kinesin-targeting motifs. Annotation of the RBD is based on close similarity to known RBDs in BNIP-2 homologs and Cdc42GAP. **B**. ClustalW multiple sequence alignment of human and mouse BNIP-2 splice variants, i.e., the database annotated hBNIP2 sequence [Swiss-Prot: Q12982] as well as short mBNIP-2 [Trembl: Q91VL0] and long mBNIP-2 [Swiss-Prot: O54940]. The black box highlights the P4-P9′ motif with the P4-P1 IEAD GrB cleavage motif and BNIP-2 domains indicated as in **A**.

To explore these hypotheses, we applied our degradomics technology to probe for efficient mGrB cleavage events. Next to caspase-7, and in analogy with a previous analysis probing for efficient hGrB cleavage events [8], BNIP-2 cleavage was here assigned amongst the most efficiently cleaved mGrB substrates. Members of the family of pro-apoptotic Bcl-2 and adenovirus E1B 19 kDa interacting proteins to which BNIP-2 belongs, are known to interact with various pathological and physiological anti-apoptotic proteins such as Bcl-2 and the adenoviral E1B 19 kDa protein [16]. In addition to several other domains, BNIP-2 holds a BNIP-2 and Cdc42GAP homology (BCH) domain (Figure 1), via which it is able to bind cdc42, a member of the Rho subfamily involved in controlling cell morphological changes, to promote the formation of cellular extensions [17] and myogenic differentiation via yet unidentified regulator(s) [18].

Besides its implication in cellular differentiation, other functions have been attributed to BNIP-2 [19], including its involvement in apoptotic signaling (for human as well as mouse BNIP-2). Massive cell death of neuroblastoma cells occurs following overexpression of hBNIP-2, and is counteracted by treatment with neuroprotective estrogen, which results in a downregulation of hBNIP-2 [20]. These findings inspired several groups to study the molecular mechanisms underlying hBNIP-2 induced apoptosis. Interestingly, *in vitro* and/or *in cellulo* cleavage of hBNIP-2 by the caspases 3, 6, 8, 9 and 10 occurs outside the BCH domain [21,22]. In addition, GrB mediated cleavage of hBNIP-2 could be observed when probing for human and mouse granzyme B substrates [1], a hGrB event furthermore predicted by a database search making use of the optimal Bid cleavage motif (i.e., IEAD) [11]. Human BNIP-2 was found to be directly processed at IEAD_28_ by granzyme B *in vitro* and during natural killer cell-mediated killing of tumor cells and thus not secondary to granzyme B induced caspase activation [11]. Although these data could suggest that caspase/granzyme-mediated release of the BCH domain influences the pro-apoptotic activity of BNIP-2, in line with the observed massive neuroblastoma cell death [20], full length hBNIP-2 by itself was able to induce cell death in HeLa cells [11].

Various signaling pathways, including activation of the intrinsic apoptotic pathway [23] or direct caspase activation [24], have been proposed to explain the apoptotic potential of BNIP-2.

Overall, we here investigated the cleavage susceptibilities of BNIP-2 - identified in this study as an efficient mGrB substrate - by the orthologous mouse and human granzymes B and demonstrated that differential substrate targeting of BNIP-2 by orthologous granzymes B is strongly influenced by the primed site residues.

## Methods

### Ethics statement

All results of this research were based on the use of cultured human (HeLa or Jurkat) or mouse (NIH/3T3 or YAC-1) cell lines. Neither human (human subjects or human derived material) nor animals (vertebrates or any regulated invertebrates) were used in this experimental research.

### Cell culture

Human HeLa cells (ATCC CCL-2, American Type Culture Collection, Manassas, VA, USA), mouse NIH/3T3 cells (ATCC CRL-1658), human Jurkat cells (ATCC TIB-152) and mouse YAC-1 cells (ATCC; TIB-160) were grown in DMEM (HeLa and NIH/3T3) or RPMI-1640 (Jurkat and YAC-1) medium, supplemented with 10% foetal bovine serum (Invitrogen, Carlsbad, CA, USA), 100 units/ml penicillin (Invitrogen) and 100 μg/ml streptomycin (Invitrogen). For proteome analyses, Jurkat cells were grown in RPMI 1640 medium (Invitrogen, Carlsbad, CA, USA, #61870-010) containing either natural, ^13^C_6_ or ^13^C_6_^15^N_4_ L-arginine (Cambridge Isotope Labs, Andover, MA, USA) at a concentration of 230 μM (i.e., 20% of its suggested concentration in RPMI at which L-arginine to proline conversion was not detectable). Media were supplemented with 10% dialyzed foetal bovine serum (Invitrogen, 26400–044), 100 units/ml penicillin (Invitrogen) and 100 μg/ml streptomycin (Invitrogen). Cells were cultured at 37°C and in 5% CO_2_ for at least six population doublings to ensure complete incorporation of the labeled arginine.

### Preparation of cell lysates for N-terminal COFRADIC analysis

SILAC-labeled Jurkat cells were pre-incubated with the pan-caspase inhibitor N-benzyloxycarbonyl-Val-Ala-Asp-fluoromethyl ketone (z-VAD-fmk) at a final concentration of 50 μM for 15 min at 37°C, washed in D-PBS and re-suspended at 7 × 10^6^ cells per ml in 50 mM Tris–HCl pH 8.0 and 100 mM NaCl. Cells were lysed by three rounds of freeze-thawing. Supernatants, cleared by centrifugation for 10 min at 16,000 g, were treated with 200 nM of mouse recombinant granzyme B (mGrB) for the indicated time points at 37°C. Recombinant mGrB was produced in *Pichia pastoris* as described previously [3]. Solid guanidinium hydrochloride was added to a final concentration of 4 M in order to inactivate mGrB and denature all proteins. Before mixing equal amounts of samples, proteins were reduced and alkylated using TCEP.HCl (1 mM final concentration (f.c.)) and iodoacetamide (2 mM f.c.) respectively, for 1 h at 30°C. Subsequent steps of the N-terminal COFRADIC protocol were performed as described previously [25]. The proteome was digested overnight at 37°C with sequencing-grade, modified trypsin (Promega, Madison, WI, USA) (enzyme/substrate of 1/100 w/w).

### LC-MS/MS analysis and data processing

ESI LC-MS/MS analysis was performed on an ESI-Q-TOF Premier (Waters Corporation) mass spectrometer as described before [26]. ESI-Q-TOF MS/MS peptide fragmentation spectra were converted to pkl files using the Masslynx® software (version 4.1, Waters Corporation). N-terminal peptides were identified using a locally installed version of the MASCOT database search engine version 2.2.1 and the Swiss-Prot database (version 13.0 of UniProtKB/Swiss-Prot protein database, containing in total 356,194 sequence entries from which 18,609 human entries) was searched with restriction to human proteins. The following search parameters were used. Peptide mass tolerance was set at 0.2 Da and peptide fragment mass tolerance at 0.1 Da with the ESI-QUAD-TOF as selected instrument for peptide fragmentation rules for the Q-TOF Premier data. Endoproteinase Arg-C/P (i.e., no restriction towards arginine-proline cleavage) was set as enzyme allowing one missed cleavage. Variable modifications were pyroglutamate formation of N-terminal glutamine, pyrocarbamidomethyl formation of N-terminal alkylated cysteine, deamidation of asparagine, acetylation and tri-deuteroacetylation of the alpha-N-terminus. Fixed modifications were methionine oxidation (sulfoxide), carbamidomethylation of cysteine, tri-deuteroacetylation of lysine and, for identifying heavy labelled peptides, [^13^C_6_] or [^13^C_6_^15^N_4_]-arginine were additionally set as fixed modifications. Only peptide-to-spectrum matches with MASCOT ion scores that exceeded the corresponding MASCOT identity threshold score (at 95% confidence level) and that were ranked one, were withheld. In addition, spectra that received a low MASCOT ion score (5 or less points above threshold for identity) were further interrogated and only spectra that contained b- and y- fragment ions covering a stretch of at least three consecutive amino acids were considered identified. The estimated false discovery rate by searching decoy databases was typically found to lie between 2 and 4% on the spectrum level [27]. Whenever a peptide matched to multiple members of a protein family (redundancy), the protein entry reported (column ‘Protein description’, Additional file 1: Table S1) was according to its alphabetical ranking. In each case, other matching protein entries were reported under the column ‘Isoforms’. The peptide intensity ratios were manually calculated from the MS data in the Masslynx® software version 4.1 environment. In order to correct for small variations in initial protein concentrations, the ^12^C_6_/^13^C_6_ and ^12^C_6_/^13^C_6_^15^N_4_ ratios of the 541 database-annotated protein N-terminal peptides identified (peptides starting at position 1 or 2) were first subjected to robust statistics using the base-2 logarithms of their ratios. The corresponding ratio means, −0.003 (^12^C_6_/^13^C_6_) and 0.009 (^12^C_6_/^13^C_6_^15^N_4_), were used to correct for the calculated ratios of all peptides identified. R was used to calculate the probability distributions of log2 transformed N-terminal peptide ratios between the 1st and 2nd time point (*N(−0.003, 0.1451) (mean, standard deviation)*) and between the 2nd and 3rd time point (*N(0.009, 0.1871)*). Protease substrate intensity ratios falling within the 98% probability interval of the protein N-terminal peptide intensity ratios were considered as derived from efficiently cleaved sites (see Results section and [8]). The mass spectrometry proteomics data have been deposited to the ProteomeXchange Consortium (http://proteomecentral.proteomexchange.org) via the PRIDE partner repository [28] with the dataset identifier PXD000204 and DOI 10.6019/PXD000204.

### Immunoblot analysis

PBS washed Jurkat cells were re-suspended in 50 mM Tris.HCl pH 8.0 and 100 mM NaCl and subjected to three rounds of freeze thaw lysis. Supernatants, cleared by centrifugation for 10 min at 16,000 g, were pre-treated with 5 mM iodoacetamide for 15 min in the dark to avoid downstream granzyme B mediated caspase activation [29]. Protein samples were subsequently treated with varying concentrations of hGrB or mGrB (ranging from 0.97 nM to 1 μM). Following GrB incubation, sample loading buffer was added and equal amounts of protein (50 μg/sample; protein concentrations were measured using the DC Protein Assay Kit from Bio-Rad (Munich, Germany)) were separated on 4-12% or 12% polyacrylamide Criterion XT-gels in MOPS buffer (Bio-Rad) at 150 V. Subsequently, proteins were transferred onto a PVDF membrane. To detect endogenous Bid and BNIP-2 proteins, membranes were blocked for 30 min in 5% skimmed milk powder containing Tris-buffered saline and 0.1% Tween-20 (TBS-T) and probed for 1 h with primary antibody in 5% skimmed milk powder in TBS-T. Following three 10 min washes in TBS-T, membranes were incubated with species-specific HRP-conjugated antibody for 1 h in 5% skimmed milk powder containing TBS-T. Again following a 3-fold wash step in TBS-T or TBS (last wash step), bands were visualized on ECL Hyperfilms (Amersham Biosciences, Buckinghamshire, UK) using an enhanced chemiluminiscence kit (PerkinElmer Life Sciences, Boston, MA, USA). Bid antibody was from R&D systems (AF860) and BNIP-2 antibody from Hypromatrix (HM1251).

### Assessing BNIP-2 peptide substrate cleavage

An oligopeptide holding the hBNIP-2 GrB cleavage site (italics) identified using N-terminal COFRADIC, NH_2_.LPEDDS*IEAD*ILAITGY(NO_2_)R.OH, was synthesized in-house using Fmoc-chemistry on an Applied Biosystems 433A Peptide Synthesizer. Cleavage progression was assayed for 2 h at 37°C with various concentrations of human and mouse GrB (50, 200 and 500 nM) at a substrate concentration of 100 μM. The reactions were stopped by adding trifluoroacetic acid to a final concentration of 1%. Cleavage by human and mouse GrB was assessed by monitoring the UV absorbance signal (214 nm) of the precursor peptide and its fragments upon reverse-phase HPLC separation. The extent of hydrolysis was determined from the intensity of the product peak(s) compared to the intensity of their unprocessed precursor.

### Granzyme cleavage of *in vitro* transcribed and translated BNIP-2

Vectors encoding wild type BNIP-2 (with or without 5′ leader) and BNIP-2 variants were used as templates for *in vitro* coupled transcription/translation in a rabbit reticulocyte lysate system according to the manufacturer’s instructions (IVTT; Promega) to generate [^35^S] methionine-labeled BNIP-2 (variants). The TnT Quick Master Mix (40 μl) (pOTB7-hBNIP-2 (RZPD Imagenes, cat n° IRAUp969D059D, Germany) and pCMV-SPORT6-mBNIP-2 (RZPD Imagenes, cat n° IRAVp968F0522D, Germany)) – consisting of a rabbit reticulocyte lysate, reaction buffer, SP6 or T7 RNA polymerase, an amino acid mixture without methionine and RNasin Ribonuclease Inhibitor – was mixed with 2 μl [^35^S]-methionine (20 μCi/ml), 1 μg of plasmid DNA template (1 μg/μl) and 7 μl nuclease-free water. Following 90 min incubation at 30°C, translates were incubated with iodoacetamide (f.c. 5 mM) for 15 min at 37°C in the dark to avoid downstream caspase activation upon incubation with granzyme B [30]. The translates (5 μl) were subsequently incubated for 1.5 h at 37°C in granzyme assay buffer with varying concentrations of hGrB or mGrB (ranging from 0.97 nM to 1 μM) in a total volume of 30 μl. Cleavage reactions were stopped by the addition of NuPAGE® LDS Sample Buffer (Invitrogen) and by heating the samples for 10 min at 70°C. Proteins were separated on 4-12% NuPAGE® Bis-Tris gradient gels and subsequently transferred onto a PVDF membrane as indicated above, air-dried and exposed to a film suitable for radiographic detection (ECL Hyperfilms, Amersham Biosciences, Buckinghamshire, UK). The percentage of cleavage was determined by densitometry following autoradiographic analysis. Quantification was performed using the ChemiGenius imaging system (SynGene, Cambridge, United Kingdom) and signal intensity was quantified using GeneTools 3.07 image analysis software (SynGene).

### Cloning of wild type BNIP-2 and BNIP-2 variants

Of note, the UniProtKB database contains entries of two mouse BNIP-2 splice variants (Swiss-Prot O54940 (long mouse BNIP-2) and Trembl Q91VL0 (short mouse BNIP-2)) which only differ by 12 additional amino acid residues at the C-terminus (Figure 1B). All experiments conducted however (unless indicated otherwise) made use of the short mBNIP-2 variant as this represents the closest hBNIP-2 ortholog (Figure 1B). The pOTB7-hBNIP-2, the pCMV-SPORT6-mBNIP-2 (short mouse BNIP-2) and the pFLCI-mBNIP-2 (long mouse BNIP-2; RZPD Imagenes, cat n° E330010L14) vectors served as templates to generate attB-flanked PCR products of full-length and mutated BNIP-2 variants suitable for use in a Gateway® BP recombination reaction with a donor vector (pDONR221, Invitrogen, cat n° 12536–017) thereby creating an entry clone. Forward primers of human, short mouse and long mouse BNIP-2 (with or without 5′ leader) and their respective reverse primers used to generate attB flanked PCR products are indicated in Additional file 2: Table S2. Of note, short and long mBNIP-2 reverse primers are identical and therefore annotated as mBNIP-2 reverse primer. Additional forward primers were designed to mimic the GrB cleaved C-terminal human and mouse BNIP-2 fragment (i.e., truncated h/m BNIP-2 or tBNIP-2), however additionally encoding an extra N-terminal Met residue in order for translation (initiation) to proceed, thereby giving rise to a tBNIP-2 with a Met-Ile- starting protein N-terminus where Ile represents the P1′ Ile_29_ of GrB cleaved full-length BNIP2_._ The reverse primers were designed to fuse the desired PCR products in frame with the C-terminal V5/His-tag encoded by the destination vector. To create BNIP-2 expression clones, the BNIP-2 inserts of the entry vectors were recombined into the pEF-DEST51 destination vector (Invitrogen, cat n° 12285–011) using LR-clonase (Invitrogen, cat n° 11791–020) according to the manufacturer’s instructions.

### Site-directed PCR-mutagenesis

Plasmids encoding the human to mouse specific mutations: I_29_ > T, A_31_ > D_,_ I_32_ > G, G_34_ > D_,_ E_36_ > D and D_37_ > R as well as combined mutants of hBNIP-2 were generated by site-directed PCR-mutagenesis (QuickChange, Stratagene) according to the manufacturer’s instructions using primer pairs listed in Additional file 2: Table S2. To create the hBNIP-2 mutant in which P1′, P6′ and P8′ are mutated, the hBNIP-2 P1′ mutant was used as template and forward and reverse primer pairs were created to generate this combined mutant (Additional file 2: Table S2; combined 1). The hBNIP-2 mutant where all differential amino acids C-terminal to the cleavage site were mutated to the corresponding amino acids present in the murine BNIP-2 ortholog, also made use of the P1′ mutant as template. In first instance, primer pairs were developed as to generate a combined P1′, P3′, P4′, P6′ and P8′ mutant (Additional file 2: Table S2; combined 2) upon which an additional PCR reaction was conducted to mutate P9′ (Additional file 2: Table S2; combined 3). In human and mouse (both short and long) BNIP-2 expression plasmids, next to mutation of the database annotated AUG translation sites, mutations in their 5′ leader sequence at the upstream non-cognate (non-AUG) translation initiation sites (uTIS) reported in [31,32] were created to monitor BNIP-2 N-terminal isoform production (Additional file 2: Table S2). The correctness of all (mutant) cDNA sequences generated was confirmed by DNA sequencing.

### BNIP-2 expression

Human HeLa and mouse NIH/3T3 cells seeded one day prior to transfection at 50 × 10^3^ cells/well in a 12-well plate, were transiently transfected for 24 h with 0.6 or 0.8 μg/ml (final concentration) of the eukaryotic expression vectors using Fugene HD (Roche; cat. n° 04709705001) according to the manufacturer’s instructions. After 28 h, cells were lysed on-plate using 80 μl lysis buffer (50 mM Tris–HCl (pH 7.8), 150 mM NaCl, 1% NP-40 and protease inhibitor cocktail tablet (Roche, cat n° 11697498001)), subjected to three rounds of freeze-thaw lysis and centrifuged for 10 min at 16,000 g. Sample loading buffer was then added and proteins were separated using 4‒12% polyacrylamide Criterion XT‒gels (1.0 mm thick gels) in MOPS buffer (Bio‒Rad) at 150 V. Subsequently, proteins were transferred onto a PVDF membrane. Membranes were blocked for 30 min in a 1:1 Tris‒buffered saline and 0.1% Tween‒20 (TBS‒T) Odyssey Blocking solution (LI-COR, cat n° 927–40003) and probed for 1 h with primary anti-V5 antibody (Invitrogen, cat n° R960-25, dilution: 1/5000) in TBS‒T/Odyssey blocking buffer. Following three 10 min washes in TBS‒T, membranes were incubated with secondary anti-mouse antibody (IRDye 800 CW goat anti-mouse antibody IgG, LI-COR, cat n° 926–32210) for 1 h in TBS‒T/Odyssey blocking buffer followed by 3 washes in TBS‒T or TBS (last wash step), bands were visualized using an Odyssey infrared imaging system (LI-COR).

### SLO mediated delivery of GrB

24 h before streptolysin-O (SLO)/GrB incubation, HeLa and NIH/3T3 cells, plated one day before transfection at 50 × 10^3^ cells/well in a 12-well plate, were transfected with 0.6 and 0.8 μg BNIP-2 as described above. After 24 h, cells were washed twice with serum-free and antibiotics-free DMEM medium and pretreated with 50 μM z-VAD-fmk for 15 min at 37°C to inhibit caspase activation. Subsequently, cells were incubated with a sublytic concentration of SLO (250 ng/ml) (as determined by flow cytometry; data not shown) and 200 nM GrB for 30 min at 37°C. After 30 min, an equal volume of DMEM containing 20% FCS (f.c. 10%) without antibiotics was added to inhibit SLO and cells were allowed to recover for 1 h. Next, cells were lysed in SDS-PAGE loading buffer (60 mM Tris pH 6.8, 10% glycerol and 2% SDS) and the lysates cleared by centrifugation for 10 min at 16,000 g. Protein samples were further processed as described above for immunoblot analysis and BNIP-2 was visualized using an anti-V5 antibody (Invitrogen, cat n° R960-25; 1/5000 dilution).

## Results

### Mouse granzyme B kinetic degradomics

Screening the degradome of mGrB in function of time allows classifying substrate cleavage events according to the efficiency by which they get cleaved [8]. Here, three freeze-thaw Jurkat cell lysates differentially SILAC (Stable Isotope Labeling by Amino acids in Cell culture [33]) labeled by either normal (^12^C_6_), ^13^C_6_ or ^13^C_6_^15^N_4_ L-arginine were incubated with 200 nM mGrB for 10, 30 and 60 min respectively, as this allows monitoring the dynamics of mGrB generated neo-N-termini. Overall, this analysis resulted in the identification of 2,312 MS/MS spectra linked to 800 N-terminal peptides. Of these, 556 (or 70%) represent database annotated protein N-termini (start position 1 or 2 and N-terminally modified with a(n) (trideutero)acetyl group), while 199 N-termini (or 25%), originating from 191 proteins hinted to proteolytic events, potentially generated by mGrB, as these did not map to position 1 or 2 while carrying a trideuteroacetyl moiety at their N-terminus. The remaining 45 N-termini (or 5%) might point to alternative translation initiation events due to the presence of a co-translationally added N-terminal acetyl moiety [34]. To assign mGrB-specific cleavage events, all neo-N-termini were compared to a previous compilation of GrB cleavage events, the latter additionally making use of an internal control setup (i.e., a setup where no GrB was added) [1]. As a result, 37 mGrB cleavage sites in 36 human proteins could unequivocally be assigned.

**Figure 2:**
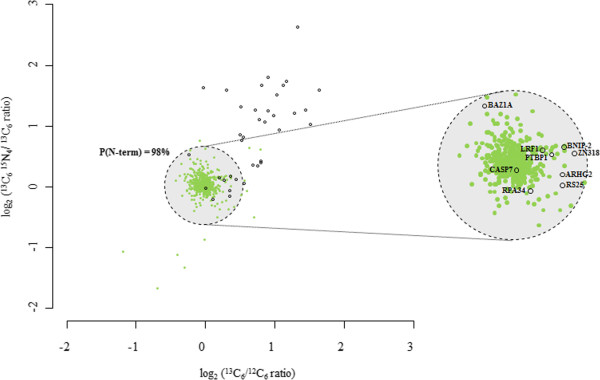
**Efficiency classification of mGrB cleavage events.** The distribution of 541 protein N-termini (green circles) and 37 mGrB specific neo-N-termini (black circles) of which the ^13^C_6_ /^12^C_6_ and ^13^C_6_^15^N_4_/^13^C_6_ ratios could be calculated, are plotted. The spreading of the values for the unaffected protein-N-termini was used to set the area containing the most efficiently cleaved mGrB sites (p ≤ 0.02). UniProtKB gene names are as in Additional file 1: Table S1.

In our setup, protein N-termini are generally not expected to change over time and their corresponding precursor ion intensities in MS should remain constant. As such, mGrB generated neo-N-termini with similar fold changes as compared to protein-N-termini point to efficiently cleaved substrates as the concentration of such neo-N-termini does not change upon prolonged protease incubation [8]. Neo-N-termini with increasing ion intensities in function of time can be considered as less efficiently cleaved (i.e., the concentration of such neo-N-termini increases upon prolonged protease incubation). By taking the intra-experimental variation of the 541 database (UniProtKB) annotated protein-N-termini for which the ^13^C_6_/^12^C_6_ and ^13^C_6_^15^N_4_/^13^C_6_ ratios could be calculated, a 98% confidence interval could be applied to distinguish efficient from less efficient mGrB cleavage sites (Figure 2). As a result, 9 cleavage sites were assigned efficiently cleaved as compared to 28 less efficiently cleaved (bystander) sites (Additional file 1: Table S1). In line with the hypothesis that mGrB induced apoptosis mainly proceeds via the activation of caspases [2,14], procaspase 7 appears amongst the most efficiently cleaved mGrB substrates. As expected, and in contrast to hGrB, (efficient) Bid cleavage was not observed [2-4]. Interestingly, another pro-apoptotic Bcl2-family member, BNIP-2 was found amongst the most efficiently cleaved mGrB substrates, as was the case for hGrB induced BNIP-2 cleavage [1,8]. More strikingly and in sharp contrast to the exclusive hGrB mediated cleavage of hBid [4], hBNIP-2 was found to be cleaved by mGrB at the identical and hGrB specific P4-P1 hBid motif IEAD.

### Cleavage efficiency of BNIP-2 by GrB: Biochemical characterization of (murinized) BNIP-2 by oligopeptide and substrate cleavage

**Figure 3:**
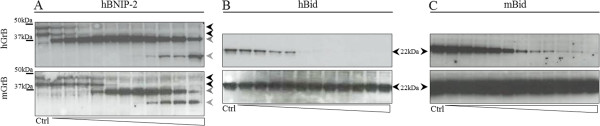
**Probing granzyme B cleavage efficiencies of Bid and BNIP-2 in cell-free lysates.** Alkylated freeze-thaw lysates of human Jurkat and mouse YAC-1 cells were incubated with serial dilutions (ranging from 1 μM to 0.97 nM (from right to left)) of human and mouse granzyme B to assess BNIP-2 (**A**: Jurkat) and Bid (**B**: Jurkat and **C**: YAC-1) cleavage. Precursors are indicated by black arrows, whereas dashed and grey arrows represent GrB generated BNIP-2 cleavage fragments. BNIP-2 results in the YAC-1 background are lacking since no endogenous BNIP-2 precursors could be detected by means of immunoblotting (data not shown).

**Figure 4:**
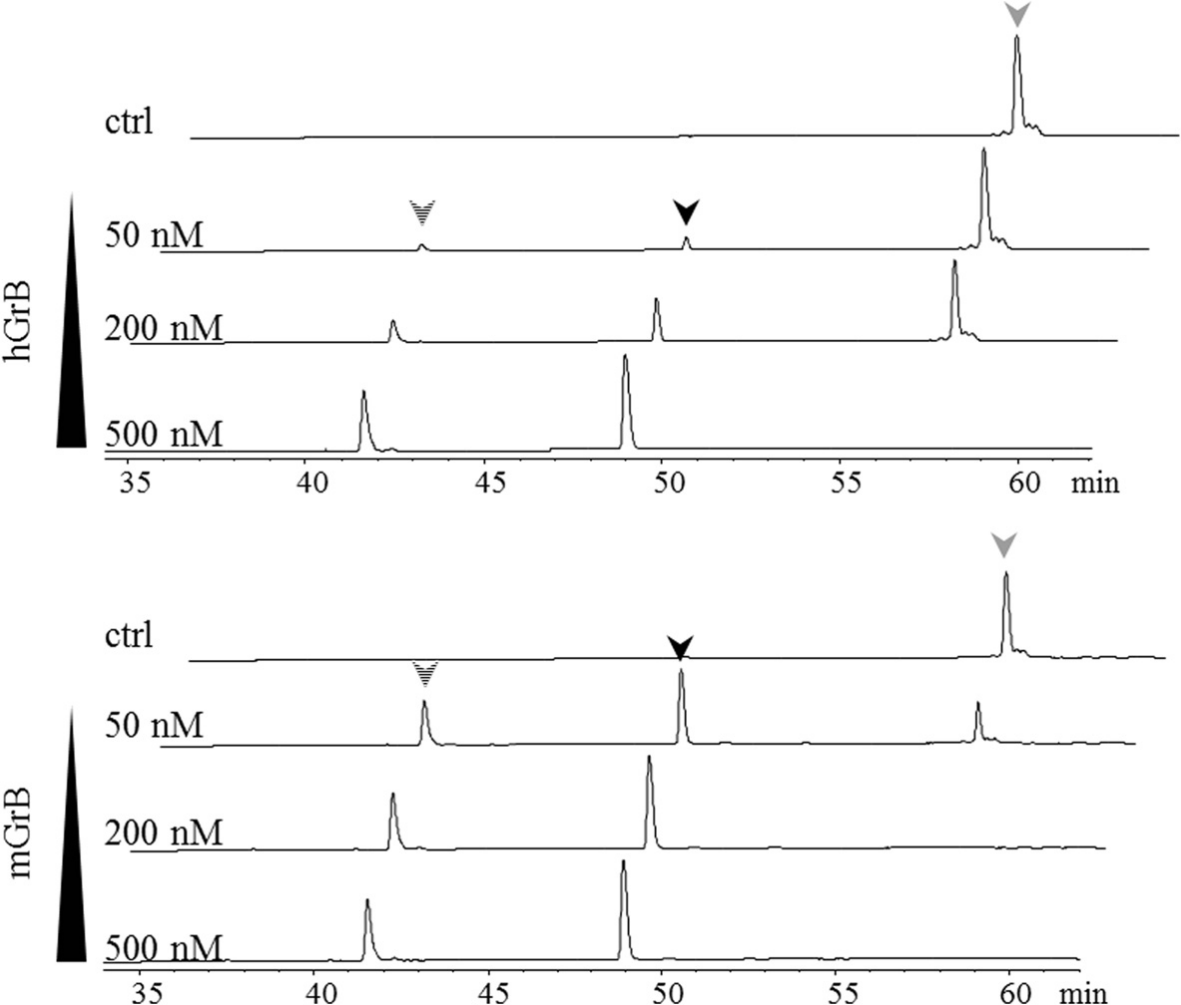
**Granzyme B mediated cleavage of a peptide substrate holding the hBNIP-2 cleavage motif.** Cleavage progression of the hBNIP-2 peptide substrate NH_2_.LPEDDSIEADILAITGY(NO_2_)R.OH was assayed for 2 h at 37°C with various concentrations of human and mouse GrB (50, 200 and 500 nM) at a substrate concentration of 100 μM, and its precursor and fragment peptides separated by RP-HPLC (chromatograms with absorbance at 214 nm are shown). The grey arrow indicates (residual) precursor peptide, whereas black and dashed arrows indicate peptide fragments generated upon human (upper panel) and mouse (lower panel) GrB mediated cleavage at IEAD (the dashed arrow corresponds to the N-terminal fragment NH_2_.LPEDDSIEAD.OH, whereas the black arrow corresponds to the C-terminal fragment NH_2_.ILAITGY(NO_2_)R.OH).

Western blot analyses using either hGrB or mGrB incubated human and mouse cell-free extracts validated our proteomics findings (Figure 3 and [8]). Cleavage of mBNIP-2 in mouse YAC-1 lysates however was not observed since BNIP-2 protein expression could not be detected even when using several BNIP-2 antibodies targeting different epitopes (data not shown). Noteworthy, both endogenous and recombinant BNIP-2 expression patterns revealed the presence of multiple BNIP-2 variants, i.e., N-terminal extended BNIP-2 variants produced by alternative translation initiation at near-cognate start codons in the 5′ leader sequence as shown below. Overall, whereas both orthologous granzymes efficiently target hBNIP-2, human and mouse Bid processing could only be observed for hGrB (Figure 3B and C). Since multiple proteolytic BNIP-2 fragments could be observed (Figure 3A) and in order to probe for GrB cleavage efficiencies at the IEAD_28_ cleavage motif identified by positional proteomics, peptides holding the hBNIP-2 P10-P6′ cleavage motif were synthesized and challenged with human or mouse GrB. Peptide cleavage analyses revealed that, while holding a hGrB specific P4-P1 hBid motif, mGrB did cleave the hBNIP-2 peptide at IEAD_28_ (Figure 4). Taking into account the greater cytotoxicity potential of hGrB versus mGrB, and the fact that so far only 16% of all cleavages identified in a proteome-wide degradomics screen were found to be uniquely or more efficiently cleaved by mGrB, mGrB cleaves the hBNIP-2 peptide about 5 times more efficient as compared to hGrB [2,3] (Figure 4).

**Figure 5:**
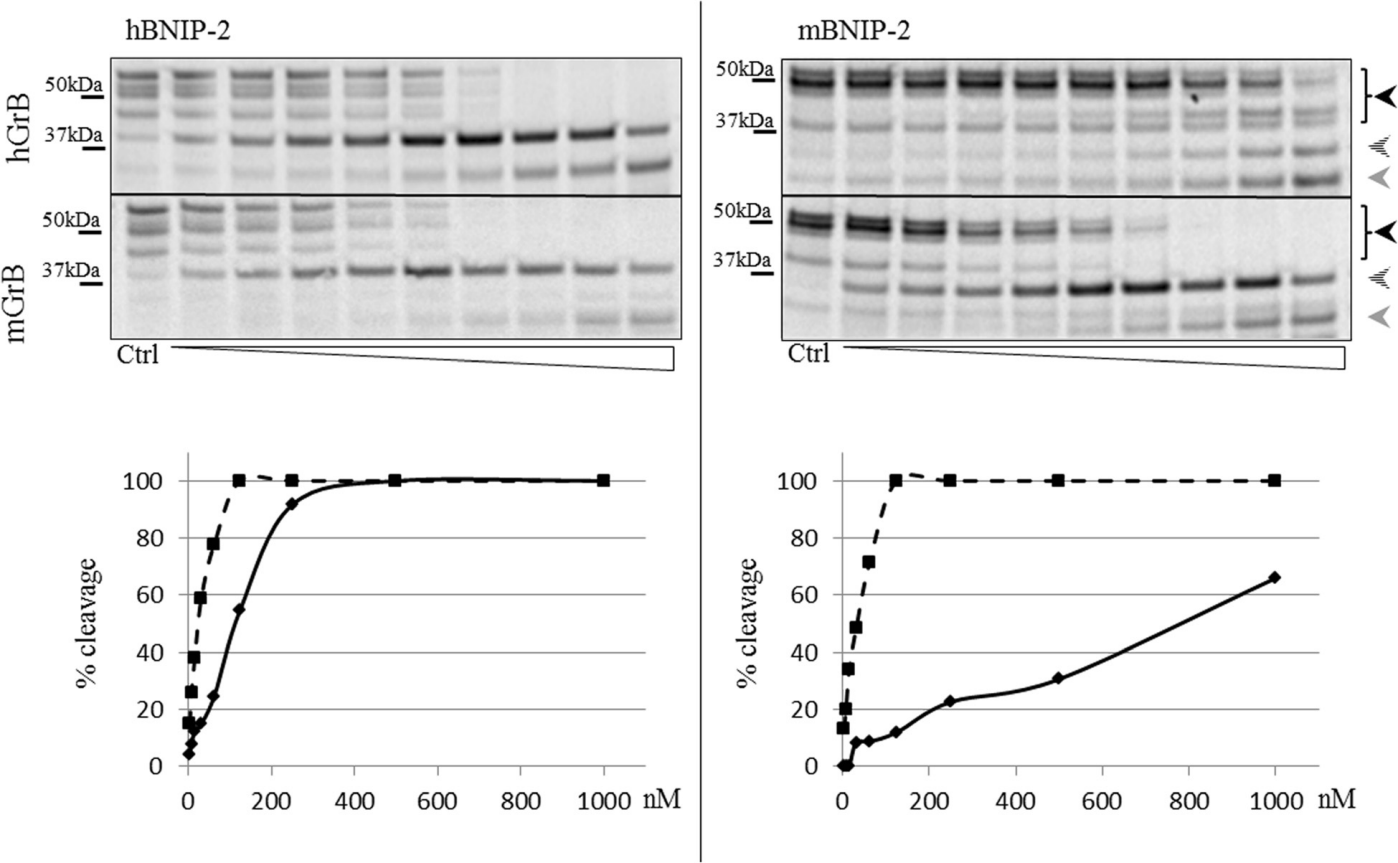
**Autoradiographs showing GrB induced cleavage of wild-type human and mouse BNIP-2.***In vitro* transcribed and translated wild-type human and mouse BNIP-2 were incubated with varying concentrations (ranging from 3.9 nM to 1 μM (from left to right)) of human and mouse GrB. Black arrows indicate BNIP-2 precursor patterns whereas dashed and grey arrows are indicative for BNIP-2 cleavage fragments. Percentages of cleavage are shown in the progression curves of hGrB and mGrB cleavage and indicated by full and dashed lines respectively.

To assess the contribution of BNIP-2 primed site residues in steering hGrB and mGrB cleavage efficiencies, (P’ mutants of) *in vitro* translated human [Swiss-Prot: Q12982] and mouse [Trembl: Q91VL0] BNIP-2 were incubated with varying concentrations of human or mouse granzyme B. In analogy to cleavage efficiencies observed when probing cell lysates, this analysis revealed efficient hBNIP-2 cleavage by hGrB and mGrB, however additionally demonstrated that mBNIP-2 was cleaved far less efficient by hGrB (± a 25-fold reduction based on the amount of protease needed to cleave 50% of the substrate), whereas both human and mouse BNIP-2 were cleaved with similar mGrB cleavage efficiencies (Figure 5). Taken together, this hints to the presence of (a) putative hGrB discriminating substrate feature(s). Interestingly, comparison of the human and mouse BNIP-2 protein sequences revealed a very high sequence similarity and identity of 96% and 93% respectively with the highest degree of divergence at the primed site sequence immediately following the scissile bond (i.e., from P1′ to P9′, next to 5 non-conservative substitutions, only 3 identical and 1 conservative amino acid substitution are found, Figure 1B).

**Figure 6:**
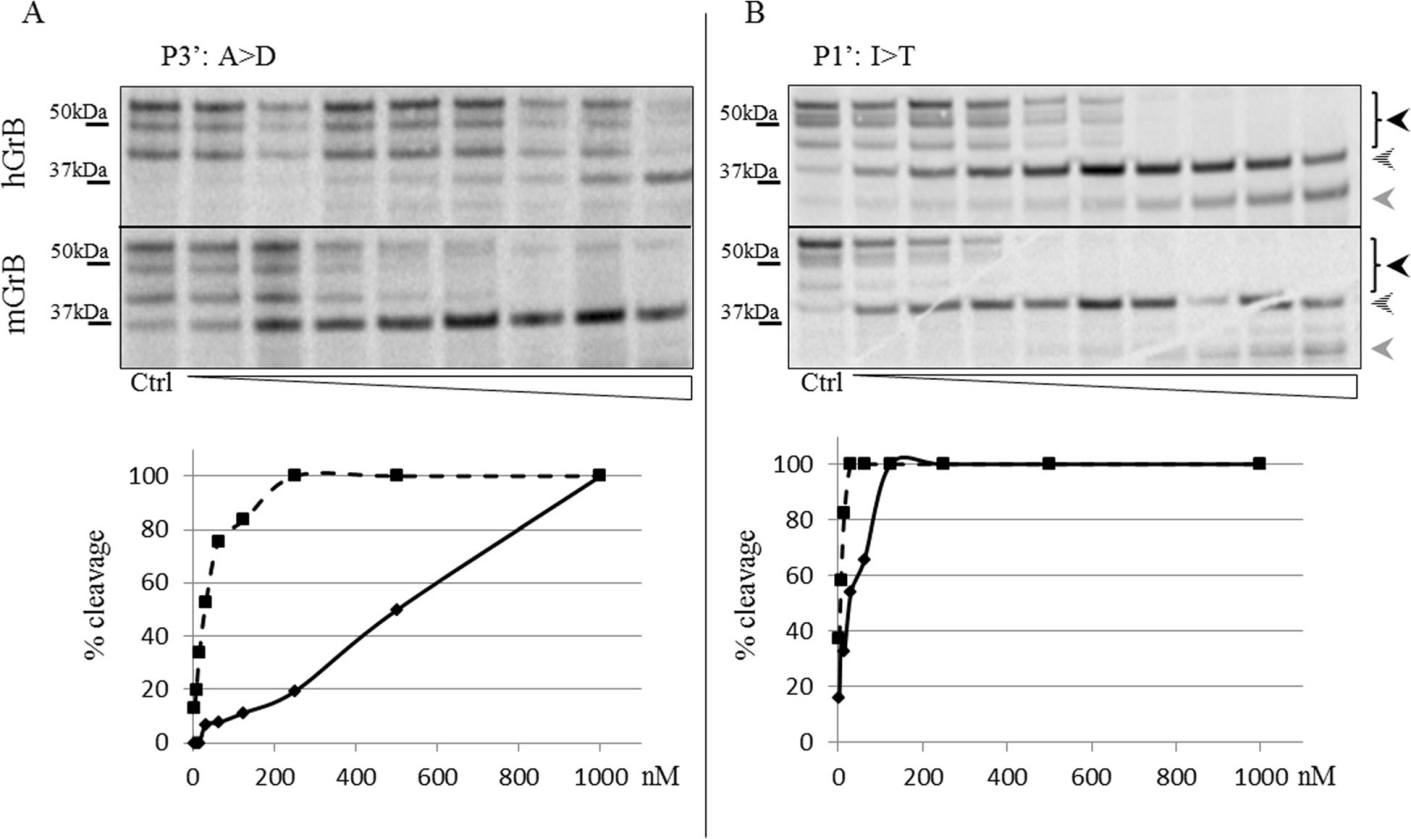
**Autoradiographs showing GrB induced cleavage of murinized human BNIP-2 variants.** P3′ **(A)** and P1′ **(B)** differing primed site residues following the identified P4-P1 cleavage site IEAD in hBNIP-2 were mutated to their corresponding mBNIP-2 amino acids. The *in vitro* transcribed and translated (mutated) BNIP-2 variants were incubated with varying concentrations (ranging from 1.95 nM to 1 μM (from left to right)) of human or mouse granzyme B. Black arrows indicate BNIP-2 precursor patterns, whereas dashed and grey arrows are indicative for BNIP-2 cleavage fragments. Percentages of cleavage are shown in the progression curves of hGrB and mGrB cleavage and indicated by full and dashed lines respectively.

**Figure 7:**
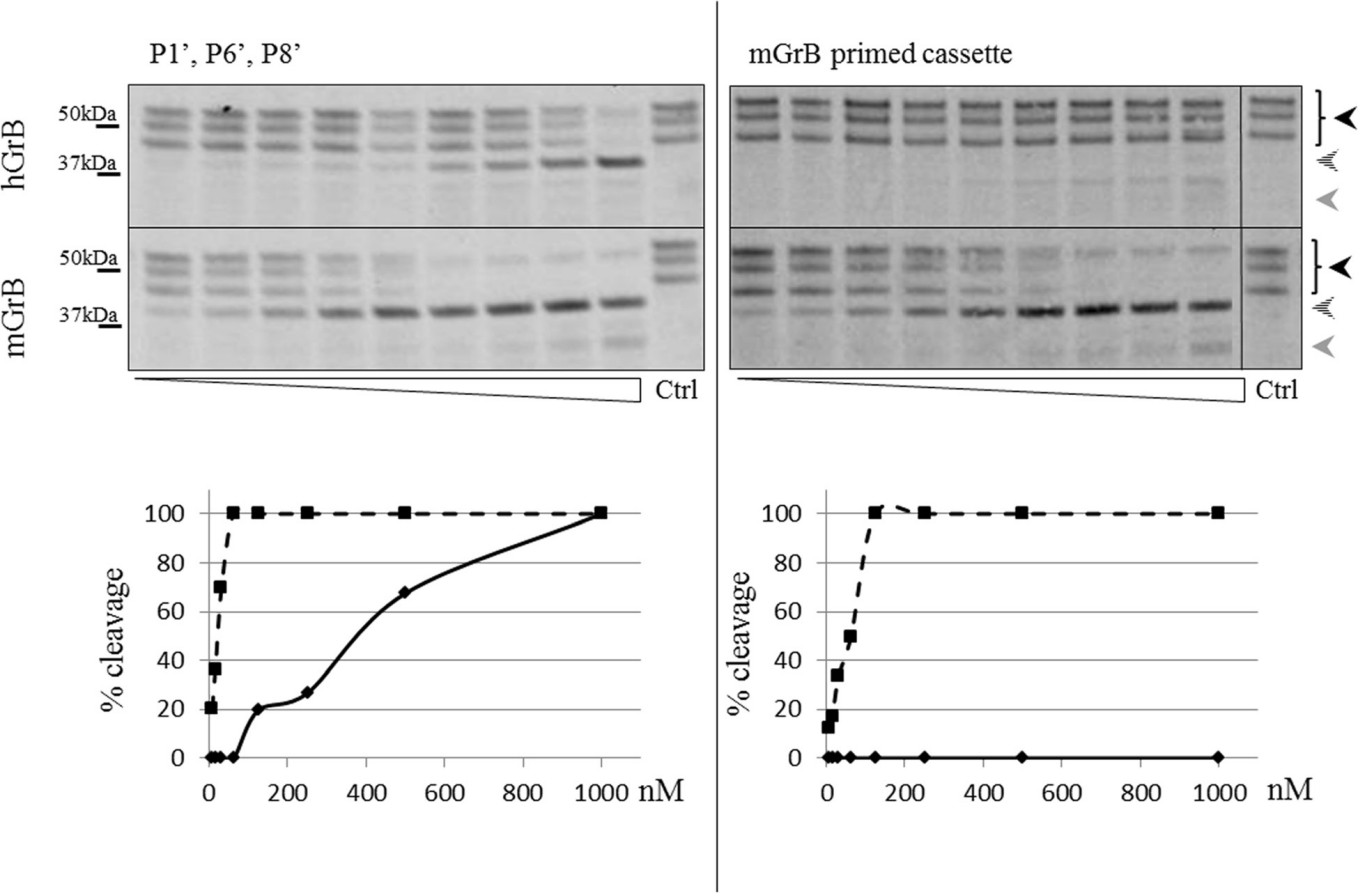
**Autoradiographs showing GrB induced cleavage of combined primed site hBNIP-2 variants.***In vitro* transcribed and translated combined hBNIP-2 variants were incubated with varying concentrations (ranging from 3.9 nM to 1 μM (from left to right)) of human and mouse GrB. Black arrows indicate BNIP-2 precursor patterns whereas dashed and grey arrows are indicative for BNIP-2 cleavage fragments. Percentages of cleavage are shown in the progression curves of hGrB and mGrB cleavage and indicated by full and dashed lines respectively.

We therefore investigated the contribution of each differing amino acid to GrB processing in the primed extended specificity profile of the IEAD_28_ motif. Using site-directed mutagenesis, we showed that the IEAA_28_ mutant was no longer processed by hGrB or mGrB (Additional file 3: Figure S1). Next, we “murinized” hBNIP-2 to investigate possible influences of the P1′-P9′ primed site motif on GrB-mediated BNIP-2 proteolysis (Figure 6 and Additional file 4: Figure S2). These analyses reveal that, although all analyzed P′ positions contribute to the differences in mGrB/hGrB cleavage efficiencies observed, the P3′ Ala_31_ to Asp mutation increases the mGrB/hGrB ratio of cleavage over 15-fold, by and large explaining the poor cleavage susceptibility of mBNIP-2 by hGrB (Figure 6A). Furthermore, cleavage of this single amino acid mutant by hGrB was about 5-fold reduced as compared to cleavage of wild-type hBNIP-2, whereas mGrB cleavage susceptibility was not affected. An Ile to Thr mutation at the P1′ position yields the highest increase in cleavage efficiency for both human and mouse GrB as this variant was cleaved about 4-fold more efficient by both orthologous granzymes as compared to wild-type hBNIP-2 (Figure 6B). In addition, mutation of P6′ and P8′ amino acids to the corresponding mouse residues both appeared favorable for cleavage by both granzymes as compared to cleavage of wild-type hBNIP-2, whereas mutation of the P9′ residue did not alter cleavage efficiency (Additional file 4: Figure S2). The creation of combined primed site mutants, such as the hBNIP-2 mutants containing the mBNIP-2 P1′, P6′ and P8′ residues, or all differentiating primed site amino acids (Figure 7), also increased the mGrB/hGrB cleavage ratio over 10-fold, indicative for the fact that multiple primed site residues contribute to the increased mGrB/hGrB cleavage ratio as compared to hBNIP-2.

### BNIP-2 cleavage upon SLO-mediated GrB delivery *in cellulo*

**Figure 8:**
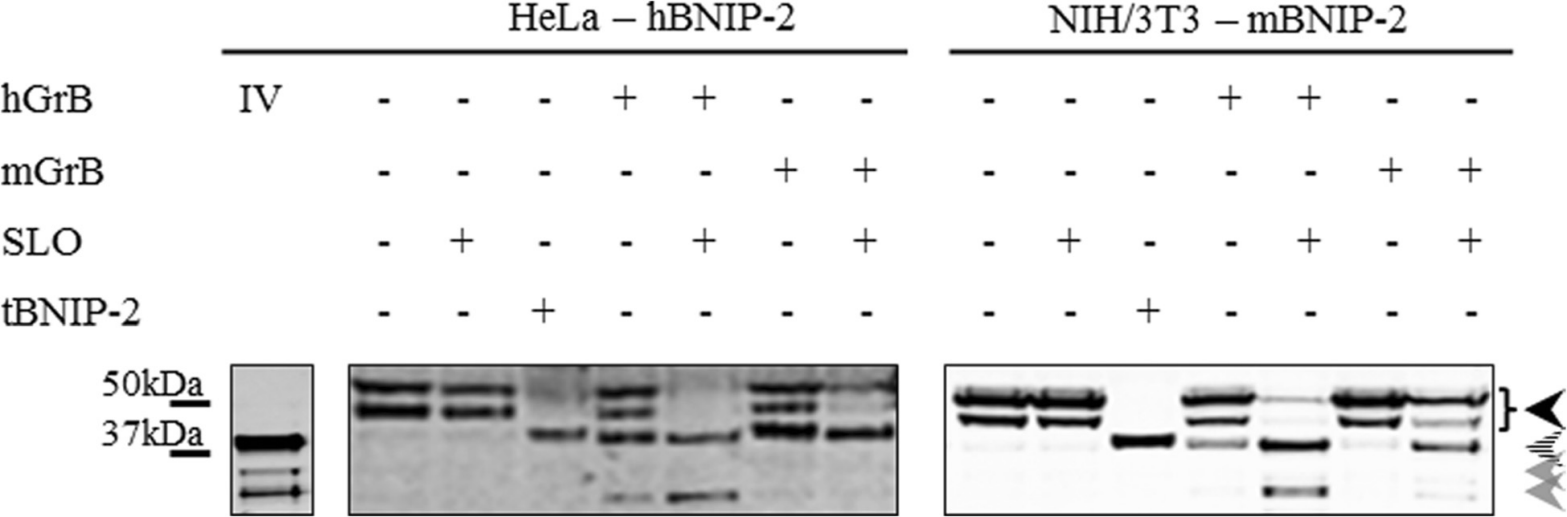
**GrB induced BNIP-2 cleavage *in cellulo*.** Human and mouse GrB delivered by means of SLO to human HeLa and murine NIH/3T3 cells transfected with hBNIP-2 and mBNIP-2 respectively were examined for *in cellulo* BNIP-2 cleavage. The *in vitro* (IV) hGrB induced cleavage pattern of hBNIP-2 in transfected HeLa cell lysates as well as HeLa and NIH/3T3 cells transfected with the truncated hBNIP-2 and mBNIP-2 variants respectively are included for comparison. Black arrows indicate BNIP-2 precursor patterns whereas dashed and grey arrows are indicative for BNIP-2 cleavage fragments.

To assess if our *in vitro* findings can be extrapolated to cell cultures and to corroborate the results reported by Scott *et al.*[11] who demonstrated cleavage of hBNIP-2 by hGrB during natural killer mediated cell death, we pursued GrB cleavage of overexpressed human and mouse BNIP-2 by means of SLO-mediated GrB delivery in HeLa and NIH/3T3 cells respectively (Figure 8). Indeed, our results show that next to human BNIP-2, overexpressed mouse BNIP-2 is efficiently cleaved during human and mouse GrB induced cell death. Transfection of truncated BNIP-2 (i.e., the GrB generated cleavage fragment (tBNIP-2)) confirms cleavage at the IEAD_28_ motif of full-length BNIP-2. However, whereas previous studies [11,23,24] reporting on the overexpression of full-length and/or truncated hBNIP-2 led to moderate poly (ADP) ribose polymerase 1 (PARP-1) cleavage and/or caspase activation, indicative for apoptosis, when transfecting (truncated) human and mouse BNIP-2 variants, we were unable to observe caspase activity or any of the typical apoptosis related hallmarks using fluorimetric caspase assays and flow cytometry (data not shown).

### Assessing the influence of 5′ leader sequences on BNIP-2 precursor patterns

**Figure 9:**
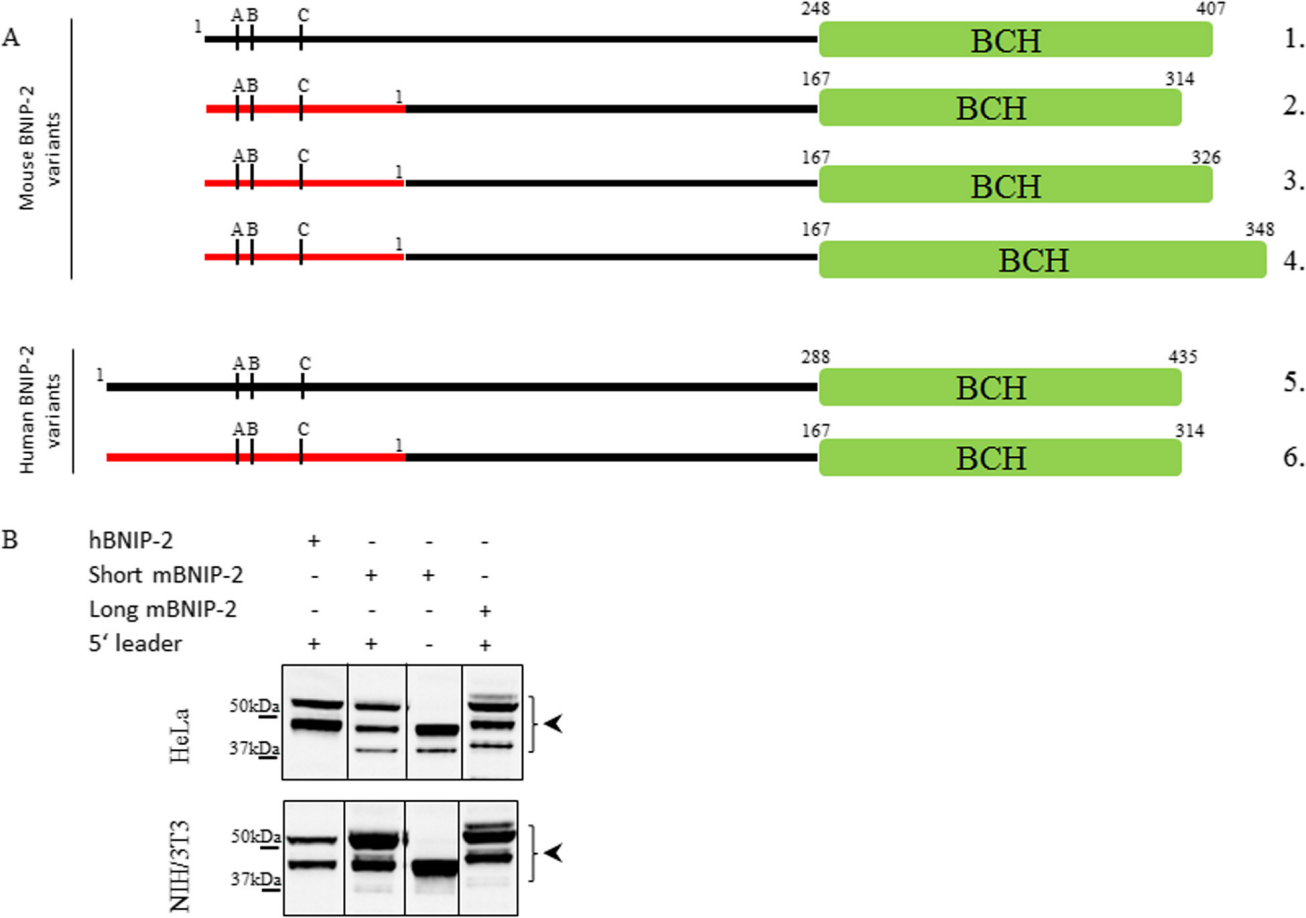
**Upstream translation initiation sites (uTIS) in the 5’ leader sequences of BNIP-2. A.** Domain architecture and BCH domains of human and mouse BNIP-2 variants. In-frame translation products possessing a BCH domain were taken into account (i.e., four mouse (1–4) and two human (5–6) BNIP-2 variants). From top to bottom: 1. UniProtKB accession long mBNIP-2 (O54940) complemented with the 5′ leader from ENSMUSG00000011958; 2. Short mBNIP-2 [Trembl: Q91VL0]; 3. Long mBNIP-2 [Swiss-Prot: O54940]; 4. G5E8U9 (Trembl), 5. J3KN59 (Trembl) and 6. Human BNIP-2 [Swiss-Prot: Q12982]. Red bars are indicative for 5′ leader sequences whereas A, B and C annotations refer to putative alternative upstream translation initiation sites deduced from ribosome profiling analyses of a human (C) and mouse (A and B) cell line. **B.** Assessing the influence of 5′ leader sequences on BNIP-2 precursor patterns. The involvement of upstream translation initiation (uTIS) in the appearance of multiple precursor bands was investigated by transfecting BNIP-2 with or without its 5′ leader sequence in human HeLa and mouse NIH/3T3 cells. Black arrows indicate BNIP-2 precursors.

In line with previous findings in various human cell lines (i.e., NK and Daudi [11]) and when probing for endogenous or *in vitro* translated BNIP-2, in each case clearly distinguishable higher molecular BNIP-2 precursor bands could be observed (Figures 3, 5, 6, 7 and 8). Previously, these BNIP-2 variants were hypothesized to result from posttranslational modifications [35] or alternative splicing, as is the case for two other BNIP-2 homologs [36]. Of note also is the presence of two mBNIP-2 splice variants in the UniProtKB database (here referred to as short [Trembl: Q91VL0] and long mouse BNIP-2 [Swiss-Prot: O54940]), only differing in 12 additional amino acids close to the C-terminus harbored in its BCH domain (Figures 1B and 9A).

**Figure 10:**
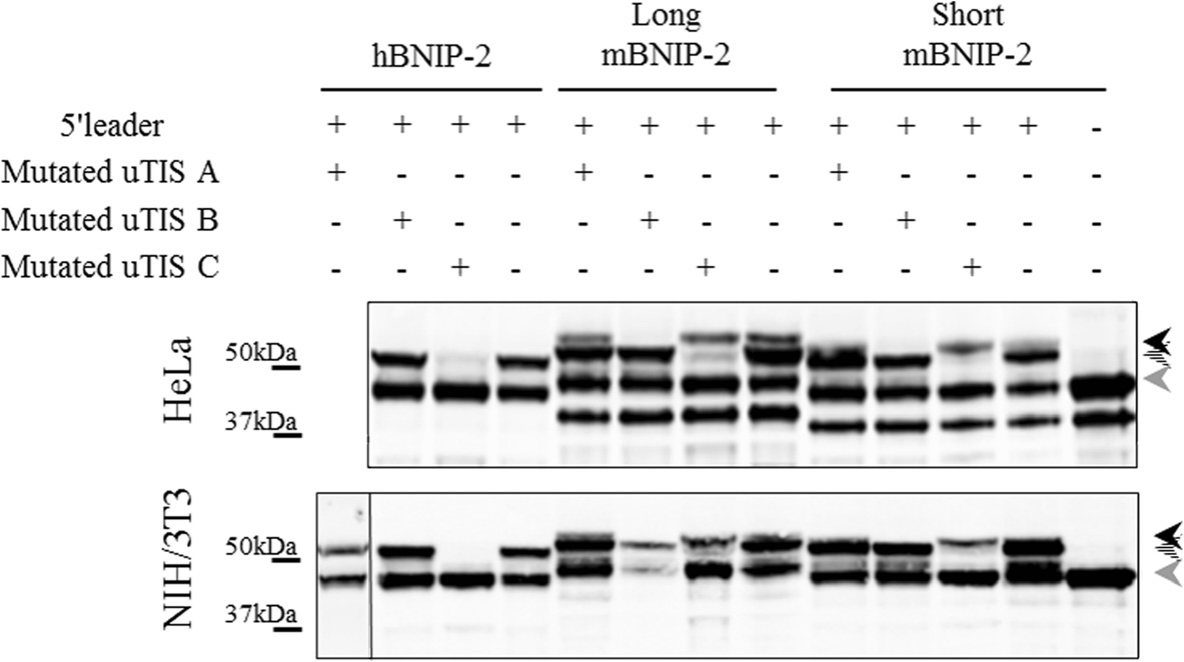
**Assessing the influence of 5′ leader sequences on BNIP-2 precursor patterns.** The contribution of alternative translation initiation to the multiple precursor bands was assessed by transfecting HeLa and NIH/3T3 cells with various uTIS mutated BNIP-2 expressing constructs. Whereas mutation of uTIS A did not alter the precursor pattern observed in the human and mouse BNIP-2 control setups, mutation of uTIS B (black arrow) resulted in the disappearance of the precursor band with the highest MW in murine BNIP-2 variants. The dashed arrow is indicative for the N-terminally extended BNIP-2 variant raised upon translation initiation at uTIS C. The BNIP-2 Swiss-Prot database annotated precursor band is annotated with a grey arrow (cfr. Additional file 7: Figure S5).

Genome-wide translation initiation profiling using Ribo-Seq [31,32] however indicates that the higher molecular weight BNIP-2 species might represent N-terminally extended BNIP-2 variants raised by alternative translation initiation at near-cognate start codons in the 5′ leader sequence or presumed 5′UTR of BNIP-2. Ribosome profiling allows sequencing of ribosome-protected mRNA fragments [31] and, combined with the use of translation inhibitors that cause accumulation of ribosomes at initiation codons, the exact translation initiation sites and ORFs can be delineated [31,32]. Genome-wide analyses of *in vivo* translation in mouse embryonic stem cells and human Hek293t cells led to the identification of respectively one human and two mouse upstream in-frame translation initiation site(s) potentially giving rise to N-terminally extended BNIP-2 variants (Figure 9A and Additional file 5 and Additional file 6: Figures S3 and S4). Since the original expression plasmids did hold part of the 5′ leader sequence (53% and 82% of the hBNIP-2 and mBNIP-2 5′ leader sequences respectively) (Additional file 5 and Additional file 6: Figures S3 and S4), we wanted to experimentally monitor translation initiation at the near-cognate start codons upstream and in-frame of the database annotated translation initiation start site (dbTIS). Therefore, we created a mBNIP-2 expression plasmid deprived of its 5′ leader sequence and examined the precursor profile upon transfection in human HeLa and mouse NIH/3T3 cells (Figure 9B). Indeed, the higher molecular weight precursor band could no longer be observed. Given that translation initiation at upstream TIS (uTIS) A (a ctg codon in hBNIP-2 and atc in both mBNIP-2 variants) and uTIS B (tgg in hBNIP-2 and ctg in both mBNIP-2 variants) or uTIS C (ctg in all BNIP-2 variants studied) in theory increases the molecular weight of the database annotated BNIP-2 by 7 kDa and 4.5 kDa respectively (Figure 9A and Additional file 5 and Additional file 6: Figures S3 and S4), these findings correlate well with the different molecular weight precursor bands observed when probing endogenous, overexpressed and *in vitro* translated BNIP-2 (Figures 3, 5, 6, 7 and 8) [11]. To investigate this in more detail, BNIP-2 translation products were assessed in HeLa and NIH/3T3 cells transfected with human and mouse BNIP-2 expression plasmids mutated at each of the putative uTIS (uTIS A-C) reported (Figure 10). For hBNIP-2, translation initiation at uTIS C (ctg) (i.e., the uTIS reported by ribosome profiling in Hek293t cells [32]) resulted in the production of an N-terminally extended BNIP-2 variant. Mutation of the most upstream putative TIS reported, uTIS A, however did not alter the precursor pattern observed in the human and mouse BNIP-2 control setups. Finally, mutation of the database annotated initiator methionine in human and mouse BNIP-2 resulted in the disappearance of the lowest molecular weight band (Additional file 7: Figure S5). Overall, these analyses unequivocally point to the use of alternative translation initiation start sites in the 5′ leader of BNIP-2 leading to the production of N-terminally extended BNIP-2 variants.

## Discussion

Our current and previous degradome analyses led to the identification of human BNIP-2, a pro-apoptotic Bcl-2 family member, as an efficient human and mouse granzyme B substrate. For mGrB however, this represents an unanticipated cleavage event given that neither human nor mouse Bid are cleaved by mGrB whereas hBNIP-2 is cleaved after the hGrB specific P4-P1 Bid tetrapeptide motif IEAD. Up till now the P4-P1 specificity profile was considered to be the main determinant for substrate recognition. In this study, hBNIP-2 was found to be efficiently processed by both orthologous granzymes B at IEAD_28_. Interestingly, at the peptide-level mGrB was found to cleave the hBNIP-2 specific peptide 5-fold more efficiently as compared to hGrB. On the other hand, hGrB appeared to process hBNIP-2 in cell-free extracts more efficiently while mGrB processing of *in vitro* translated human and mouse BNIP-2 exceeded hGrB cleavage efficiencies over 25-fold. These discrepancies in cleavage efficiencies are likely explained by structurally different accessibilities of the cleavage site.

Structural analyses using IUPred [37], SEG [38] and DisoPred [39], all predict the GrB targeted IEAD_28_ site to reside in an unstructured/disordered region of the human and mouse BNIP-2 orthologs, a feature reminiscent to the Bid IEAD_75_ motif residing in an unstructured/disordered region of the available crystal and NMR structures of Bid [40]. Strengthened by the observation that all N-terminal extended BNIP-2 variants display equal GrB cleavage susceptibilities, this implies that, if the overall accessibility of the cleavage site is similar, binding differences and thus differences in the mGrB and hGrB cleavage efficiencies must be found in the detail of the extended substrate motif recognized. Remarkably, the most noteworthy sequence context differences between the highly similar human and mouse BNIP-2 sequences were found immediately C-terminal to the scissile bond and more specifically from P1′ to P9′. While structural modelling and molecular dynamics simulation cannot reliably model the flexible N-terminus providing further molecular insight, murinization of hBNIP-2 was done to investigate the contribution of each differing amino acid to the higher cleavage efficiency observed for mGrB. Our data show the P3′ Ala to Asp mutation as the most critical determinant for the observed differences in mGrB/hGrB mediated mBNIP-2 cleavage. On the other hand, next to amino acids present in the P6′ and P8′ position, the largest increase in cleavage efficiency was obtained when mutating Ile in the P1′ position to Thr. These results are in accordance with the previously reported extended substrate specificity profiles of human and mouse GrB [1], where a preference for small residues was found at the P1′ position next to a preference for acidic residues from the P3′ position onwards, thus validating the increased cleavage susceptibility of mGrB and hGrB observed when mutating amino acids in the P1′ and P6′ positions to threonine and aspartic acid respectively. Combination of primed site mutations increased mGrB/hGrB cleavage ratios over 10-fold (as compared to wild type hBNIP-2 cleavage; i.e., P1′, P6′ and P8′ residues) hinting to primed site substrate cooperativity steering GrB cleavage. Further, substitution of all differing hGrB primed site residues following Asp_28_ by the entire murine primed cassette resulted in cleavage efficiencies approaching those observed for mGrB cleavage of mBNIP-2. Furthermore, BNIP-2 cleavage in a cellular context was shown to occur upon SLO-mediated GrB delivery, hinting to its physiological relevance.

Comparing kinetic degradomics data of human and mouse GrB obtained in an identical proteome background showed that among the efficiently targeted GrB cleavage sites identified, four were shared between mGrB (Additional file 1: Table S1) and hGrB [8]. While three out of four were found processed with similar efficiencies, human granzyme B cleavage of caspase-7 was found to proceed less efficiently. More specifically, the neo-N-terminus generated upon granzyme B cleavage (S_199_GPINDTDANPR) carries a glycine residue at the P2′ position which was previously shown by us and others [3,8] to be preferred by mGrB, and thus explains the higher efficiency of mGrB/hGrB cleavage observed by means of kinetic degradome analyses [8]. An observation in line with the assumption that mGrB induced apoptosis mainly proceeds via the activation of caspases [2,14].

Interestingly, multiple BNIP-2 precursor bands were observed for endogenous and *in vitro* translated or overexpressed BNIP-2 in HeLa and NIH/3T3 cells. The omnipresence of alternative translation initiation [41] and the recent finding of potential uTIS within the 5′ leader sequence of BNIP-2 [31,32], led us to investigate whether translation at the reported uTIS were causative for the composite BNIP-2 precursor patterns observed. Transfection of an eukaryotic mBNIP-2 expression vector depleted of the 5′ leader sequence indeed confirmed the usage of at least one uTIS leading to the creation of an N-terminally extended form of the database annotated BNIP-2 sequence, an observation further validated when overexpressing various human and mouse BNIP-2 uTIS mutants in HeLa and NIH/3T3 cells. In the current study, translation initiation at uTIS A, the most upstream uTIS determined by ribosome profiling in mice, could not be observed. This difference could well be explained by the fact that the original BNIP-2 expression constructs only hold part of the 5′ leader sequences, thus potentially lacking regulatory sequences promoting translation initiation at this site [42]. The potential importance of amino terminal BNIP-2 extensions can be exemplified by the observation that an N-terminally extended Bid splice variant has an altered subcellular localization potentially influencing cellular apoptosis [43]. Strikingly, we also observed a cell context dependency of TIS usage, the regulation of which requires follow-up studies. Next, Scott *et al.* reported that both the full-length as well as the GrB generated BNIP-2 fragments induce PARP-1 cleavage upon transfection in HeLa cells [11]. Of note however, expression of (t)BNIP-2 resulted only in a very low percentage of PARP-1 cleavage [11]. Moreover, a 24 h transfection of the empty vector or a mock control transfection clearly also resulted in PARP-1 cleavage which approximated (t)BNIP-2 induced PARP-1 cleavage. In this study, none of the techniques suitable for the detection and/or quantification of the degree of apoptosis applied, were capable of detecting evidence of cell death in BNIP-2 or tBNIP-2 transfected human (HeLa) and murine (NIH/3T3) cells while positive controls (staurosporin to induce apoptosis) did result in an apoptotic readout, questioning the (cell-type dependent) role of BNIP-2 in apoptotic signaling.

## Conclusions

The differential cleavage susceptibilities of BNIP-2 by orthologous granzymes B can be explained by the presence of different primed site residues in the mouse and human BNIP-2 orthologs, in accordance with differential subsite requirements observed for both granzymes [1-4]. Whether mBNIP-2 plays a pivotal role in mGrB induced cell death and how alternative translation initiation of BNIP-2 affects BNIP-2 function remains to be elucidated.

### Availability of supporting data

The data set supporting the results of this article is included within the article (and its additional file(s)).

## Abbreviations

(h/m)GrB: (human/mouse) granzyme B; (t)Bid: (truncated) BH3-interacting domain death agonist; AcD3: Trideutero-acetyl; aTIS: Alternative translation initiation site; BCH: BNIP-2 and Cdc42GAP homology; BH: Bcl-2 homology; BNIP-2: BCL2/adenovirus E1B 19 kDa protein-interacting protein 2; COFRADIC: Combined fractional Diagonal chromatography; dbTIS: Database annotated translation initiation site; f.c.: Final concentration; IAA: Iodoacetamide; IAP: Inhibitor of apoptosis; ORF: Open reading frame; PARP-1: Poly (ADP) ribose polymerase 1; SCX: Strong cation exchange; SILAC: Stable isotope labeling by amino acids in cell culture; TNBS: 2,4,6-trinitrobenzenesulfonic acid; uTIS: Upstream translation initiation site; UTR: Untranslated region; zVAD-fmk: N-Benzyloxycarbonyl-Val-Ala-Asp (O-methyl) fluoromethyl ketone.

## Competing interests

The authors declare that they have no competing interests.

## Authors’ contributions

PVD and KP contributed equally to this work. PVD and KP designed research; PVD, KP, GV and VJ performed research; PVD, KP and SMS analyzed data; PVD and KP wrote the paper; PVD and KG supervised research. All authors read and approved the final manuscript.

## Supplementary Material

Additional file 1: Table S1 Classification of 37 mGrB generated neo-N-termini and their corresponding substrates. The UniProtKB/Swiss-Prot database primary accession number, protein description, P10-P10′ sequence and fold change of the mGrB generated neo-N-termini are given between 10 and 30 min and between 30 and 60 min. For each category, proteins are ranked alphabetically according to their protein description. Additional information on the maximum score, identity threshold, isoforms and whether these identical cleavage sites were previously found in the N-terminal COFRADIC analyses screening for efficient hGrB cleavage sites/substrates [8] and analyses on hGrB, mGrB and mGrC respectively are listed [5]. Click here for file

Additional file 2: Table S2 List of primers used to design mutants and attB PCR flanked products. Mutated nucleotides are underlined. Click here for file

Additional file 3: Figure S1 Autoradiographs showing the resistance of human and mouse BNIP-2 IEAA_28_ mutants to GrB induced cleavage. *In vitro* transcribed and translated human and mouse BNIP-2 IEAA_28_ mutants were incubated with varying concentrations (ranging from 3.9 nM to 500 nM (from right to left)) of human or mouse GrB. Black arrows indicate BNIP-2 precursor patterns. Click here for file

Additional file 4: Figure S2 Autoradiographs showing GrB induced cleavage of murinized human BNIP-2 variants. P4′, P6′, P8′ and P9′ differing primed site residues following the identified P4-P1 cleavage site IEAD in hBNIP-2 were mutated to their corresponding mBNIP-2 amino acids. *In vitro* transcribed and translated BNIP-2 variants were incubated with varying concentrations (ranging from 1.95 nM to 1 μM (from left to right)) of human or mouse granzyme B. Black arrows indicate BNIP-2 precursor patterns, whereas dashed and grey arrows are indicative for BNIP-2 cleavage fragments. Percentages of cleavage are shown in the progression curves of hGrB and mGrB cleavage and indicated by full and dashed lines respectively. Click here for file

Additional file 5: Figure S3 ClustalW multiple sequence alignment of human and mouse BNIP-2 variants. The amino acid sequences of the longest hBNIP-2 annotated protein (annotated as hBNIP-2; UniProtKB accession [Trembl: J3KN59]) as well as the N-terminally extended uTIS variants of hBNIP2 [Swiss-Prot: Q12982] were used in the alignment. The longest mBNIP-2 variant is based on O54940 (Swiss-Prot) complemented with the 5′ leader from ENSMUSG00000011958 whereas other variants of mBNIP-2 (short mouse BNIP-2 [Trembl: Q91VL0] and long mBNIP-2 [Swiss-Prot: O54940]) are depicted together with their postulated N-terminal extensions. The black box highlights the P4-P1 IEAD recognition motif whereas the red box assigns the database annotated initiator Met of mouse and human BNIP-2 proteins. Click here for file

Additional file 6: Figure S4 Cloned sequences of human (A), short mouse (B) and long mouse (C) BNIP-2. Alternative translation initiation sites are indicated in italics. Red and green letters denote the putative upstream translation initiation sites identified by means of ribosome profiling in mouse and human cells respectively. Below each nucleotide sequence, amino acid sequences of putative translation products are given. Click here for file

Additional file 7: Figure S5 Assessing the influence of 5′ leader sequences on BNIP-2 precursor patterns. Assignment of the precursor band corresponding to the database annotated protein (human BNIP-2:[Swiss-Prot: Q12982], short mouse BNIP-2:[Trembl: Q91VL0] and long mouse BNIP-2:[Swiss-Prot: O54940]) by mutating their respective initiator methionines. A grey arrow points to the UniProtKB annotated BNIP-2 variants, while black and dashed arrows indicate the BNIP-2 variants associated with translation initiation at uTIS B and uTIS C respectively. Click here for file
