# Supplementary material for: Importance of extended protease substrate recognition motifs in steering BNIP-2 cleavage by human and mouse granzymes B
Source: BMC Biochem. 2014 Sep 10;15:21. doi: 10.1186/1471-2091-15-21 (PMC4169252; doi:10.1186/1471-2091-15-21)
Supplement: Additional file 2: Table S2 — List of primers used to design mutants and attB PCR flanked products. Mutated nucleotides are underlined. [file 1471-2091-15-21-S2.docx]

**Supplementary table S2: List of primers used to design mutants and attB PCR flanked products**. Mutated nucleotides are underlined.

| P1’ | I_29_>T | forward | 5'-accagaagatgatagtattgaagcagatacactagctataactgg-3' |
| --- | --- | --- | --- |
|  |  | reverse | 5'-ccagttatagctagtgtatctgcttcaatactatcatcttctggt-3' |
| P3’ | A_31_>D | forward | 5'-tattgaagcagatatactagatataactggaccagaggacc-3' |
|  |  | reverse | 5'-ggtcctctggtccagttatatctagtatatctgcttcaata-3' |
| P4’ | I_32_>G | forward | 5'-tgaagcagatatactagctggaactggaccagaggaccag-3' |
|  |  | reverse | 5'-ctggtcctctggtccagttccagctagtatatctgcttca-3' |
| P6’ | G_34_>D | forward | 5'-gatatactagctataactgatccagaggaccagcctggct-3' |
|  |  | reverse | 5'-agccaggctggtcctctggatcagttatagctagtatatc-3' |
| P8’ | E_36_>D | forward | 5'-ataactggaccagacgaccagcctggctc-3' |
|  |  | reverse | 5'-gagccaggctggtcgtctggtccagttat-3' |
| P9’ | D_37_>R | forward | 5'-ctagctataactggaccagagagacagcctggctcactagaagtt-3' |
|  |  | reverse | 5'-aacttctagtgagccaggctgtctctctggtccagttatagctag-3' |
| A | | forward | 5'-gatacactagctataactgatccagacgaccagcctggctcactag-3' |
|  |  | reverse | 5'-ctagtgagccaggctggtcgtctggatcagttatagctagtgtatc-3 |
| B | | forward | 5'-gatagtattgaagcagatacactagatggaactgatccagacgaccagcctggctcactagaagtta-3' |
|  |  | reverse | 5'-taacttctagtgagccaggctggtcgtctggatcagttccatctagtgtatctgcttcaatactatc-3' |
| C | | forward | 5'-ctagatggaactgatccagacagacagcctggctcactagaagtt-3' |
|  |  | reverse | 5'-aacttctagtgagccaggctgtctgtctggatcagttccatctag-3' |
| hBNIP-2 w/o 5’ leader | | forward | 5’- ggggacaagtttgtacaaaaaagcaggcttcaccatggaaggtgtggaacttaaagaagaatgg-3’ |
| hBNIP-2 + 5’ leader | | forward | 5’-ggggacaagtttgtacaaaaaagcaggcttcaccctgtgtccgggtcagctgctgccgccg-3’ |
| Truncated hBNIP-2 | | forward | 5’-ggggacaagtttgtacaaaaaagcaggcttcaccatgatactagctataactggaccagagg-3’ |
| hBNIP-2 | | reverse | 5’-ggggaccactttgtacaagaaagctgggtcctgttcatttttcggttcatcttgttttcc-3’ |
| Short mBNIP-2 w/o 5’ leader | | forward | 5’-ggggacaagtttgtacaaaaaagcaggcttcaccatggaaggtgtggagctgaaggaagaatgg-3’ |
| Short mBNIP-2 + 5’ leader | | forward | 5’-ggggacaagtttgtacaaaaaagcaggcttcaccccacgcgtccgtgcagccgcccgg-3’ |
| Truncated mBNIP-2 | | forward | 5’-ggggacaagtttgtacaaaaaagcaggcttcaccatgacactagatggaactgatccagac-3’ |
| Long mBNIP-2 + 5’ leader | | forward | 5’-ggggacaagtttgtacaaaaaagcaggcttcaccgactgaatccgggtcagctgcagccgcccg-3’ |
| mBNIP-2 | | reverse | 5’-ggggaccactttgtacaagaaagctgggtcctgctcactttttggtggttcttgttttcc-3’ |
| hBNIP-2 uTIS A | | forward | 5’-cgacgccgtaccgctccggccggg-3’ |
|  |  | reverse | 5’-cccggccggagcggtacggcgtcg-3’ |
| hBNIP-2 uTIS B | | forward | 5’-gctgcggccgggggatttagccggggtc-3’ |
|  |  | reverse | 5’-gaccccggctaaatcccccggccgcagc-3’ |
| hBNIP-2 uTIS C | | forward | 5’-cctgctttttgcgacctcgccgtcagccc-3’ |
|  | | reverse | 5’-gggctgacggcgaggtcgcaaaaagcagg-3’ |
| mBNIP-2 uTIS A | | forward | 5’-gcccggactgcaactaagctcgtccgggaaa-3’ |
|  | | reverse | 5’-tttcccggacgagcttagttgcagtccgggc-3’ |
| mBNIP-2 uTIS B | | forward | 5’-cgtccgggaaaagctcggccccgt-3’ |
|  | | reverse | 5’-acggggccgagcttttcccggacg-3’ |
| mBNIP-2 uTIS C | | forward | 5’-cgctttctgcgacctcgccgtcagcc-3’ |
|  | | reverse | 5’-ggctgacggcgaggtcgcagaaagcg-3’ |
| hBNIP-2 dbTIS | | forward | 5’-gaatccaggctgaggatcgaaggtgtggaacttaa-3’ |
|  | | reverse | 5’- ttaagttccaccaccttcgatcctcagcctggattc-3’ |
| Short mBNIP-2 dbTIS | | forward | 5’-gtctaggctgaggatcgaaggtgtggagctg-3’ |
|  | | reverse | 5’-cagctccacaccttcgatcctcagcctagac-3’ |
| Long mBNIP-2 dbTIS | | forward | 5’-gtctaggctgaggatcgaaggtgtggagctg-3’ |
|  | | reverse | 5’-cagctccacaccttcgatcctcagcctagac-3’ |
