# Supplementary figures and images for: Importance of extended protease substrate recognition motifs in steering BNIP-2 cleavage by human and mouse granzymes B
Source: BMC Biochem. 2014 Sep 10;15:21. doi: 10.1186/1471-2091-15-21 (PMC4169252; doi:10.1186/1471-2091-15-21)

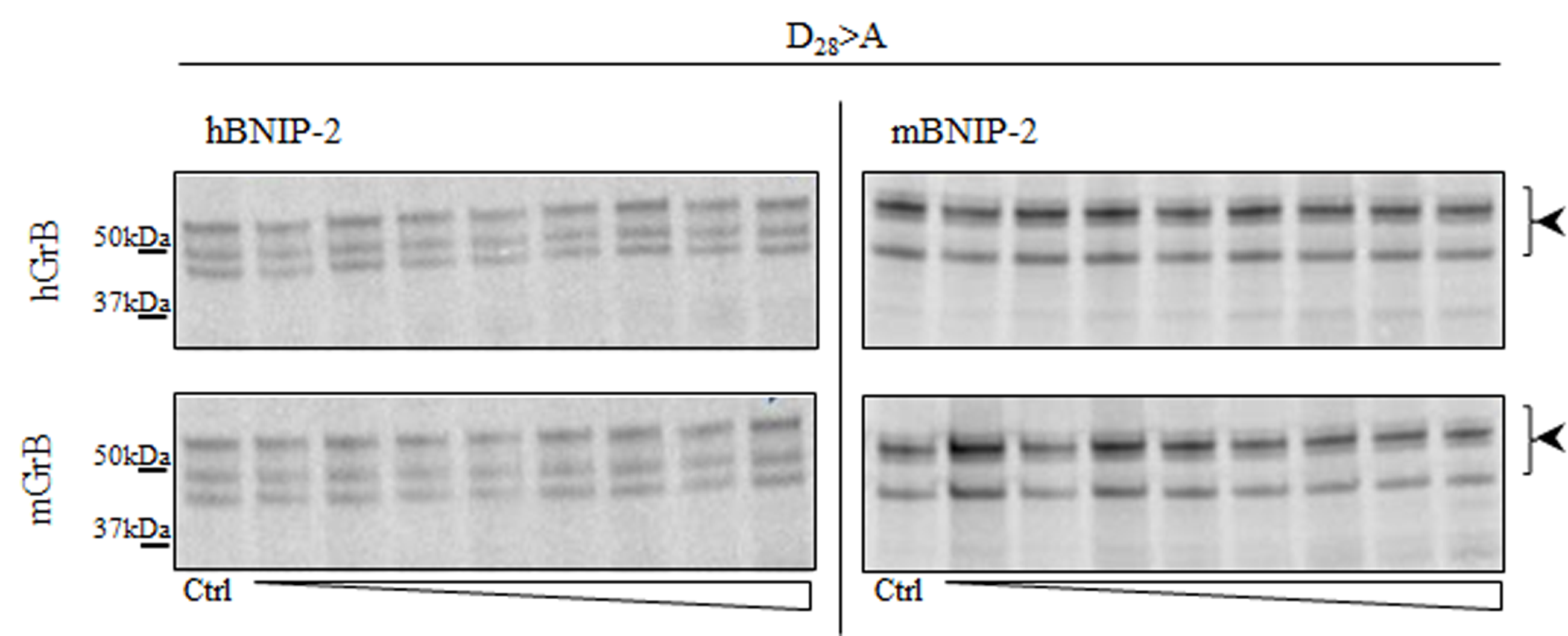

Supplement: Additional file 3: Figure S1 — Autoradiographs showing the resistance of human and mouse BNIP-2 IEAA28 mutants to GrB induced cleavage. In vitro transcribed and translated human and mouse BNIP-2 IEAA28 mutants were incubated with varying concentrations (ranging from 3.9 nM to 500 nM (from right to left)) of human or mouse GrB. Black arrows indicate BNIP-2 precursor patterns. [file 1471-2091-15-21-S3.tiff]

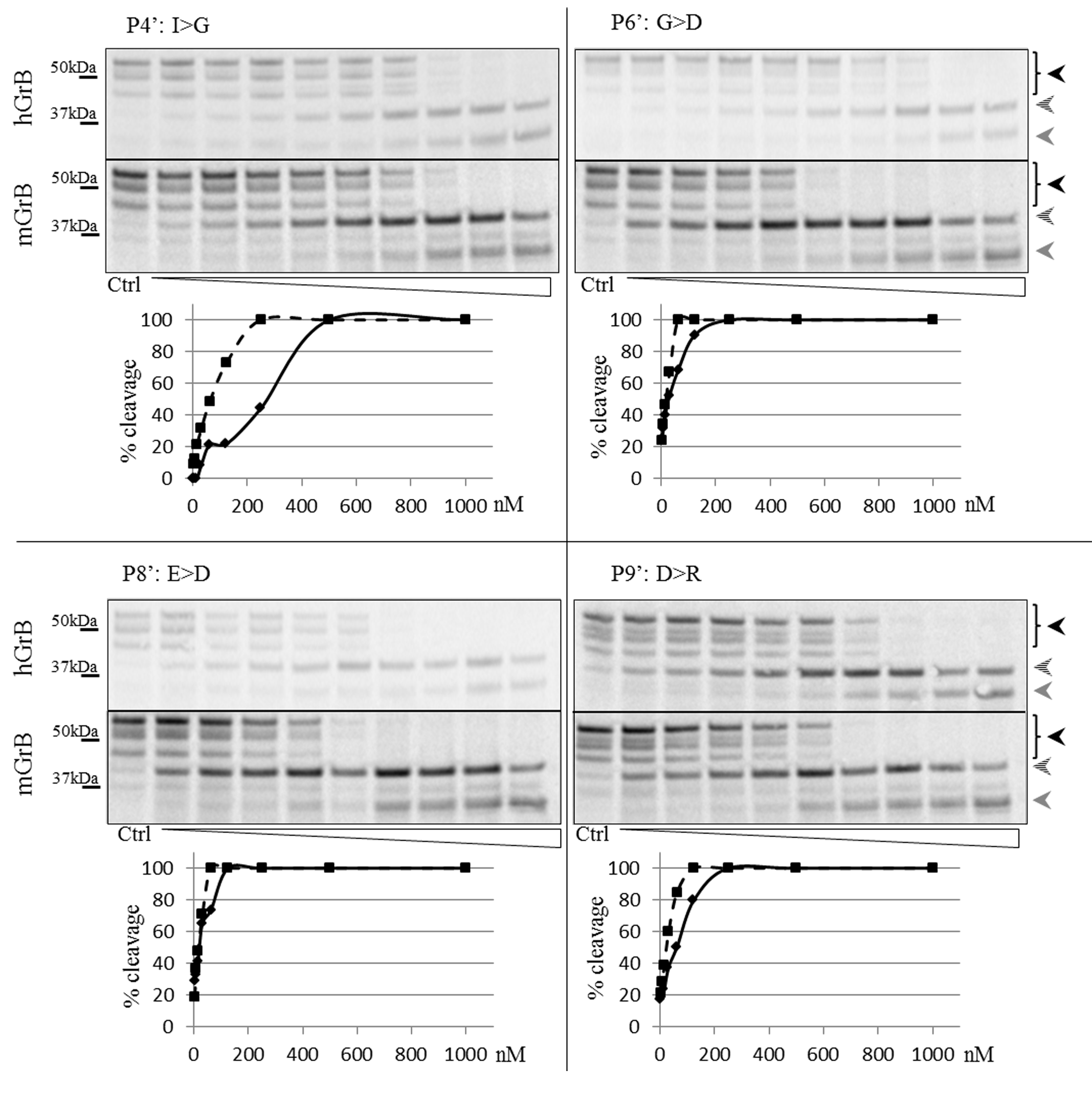

Supplement: Additional file 4: Figure S2 — Autoradiographs showing GrB induced cleavage of murinized human BNIP-2 variants. P4′, P6′, P8′ and P9′ differing primed site residues following the identified P4-P1 cleavage site IEAD in hBNIP-2 were mutated to their corresponding mBNIP-2 amino acids. In vitro transcribed and translated BNIP-2 variants were incubated with varying concentrations (ranging from 1.95 nM to 1 μM (from left to right)) of human or mouse granzyme B. Black arrows indicate BNIP-2 precursor patterns, whereas dashed and grey arrows are indicative for BNIP-2 cleavage fragments. Percentages of cleavage are shown in the progression curves of hGrB and mGrB cleavage and indicated by full and dashed lines respectively. [file 1471-2091-15-21-S4.tiff]

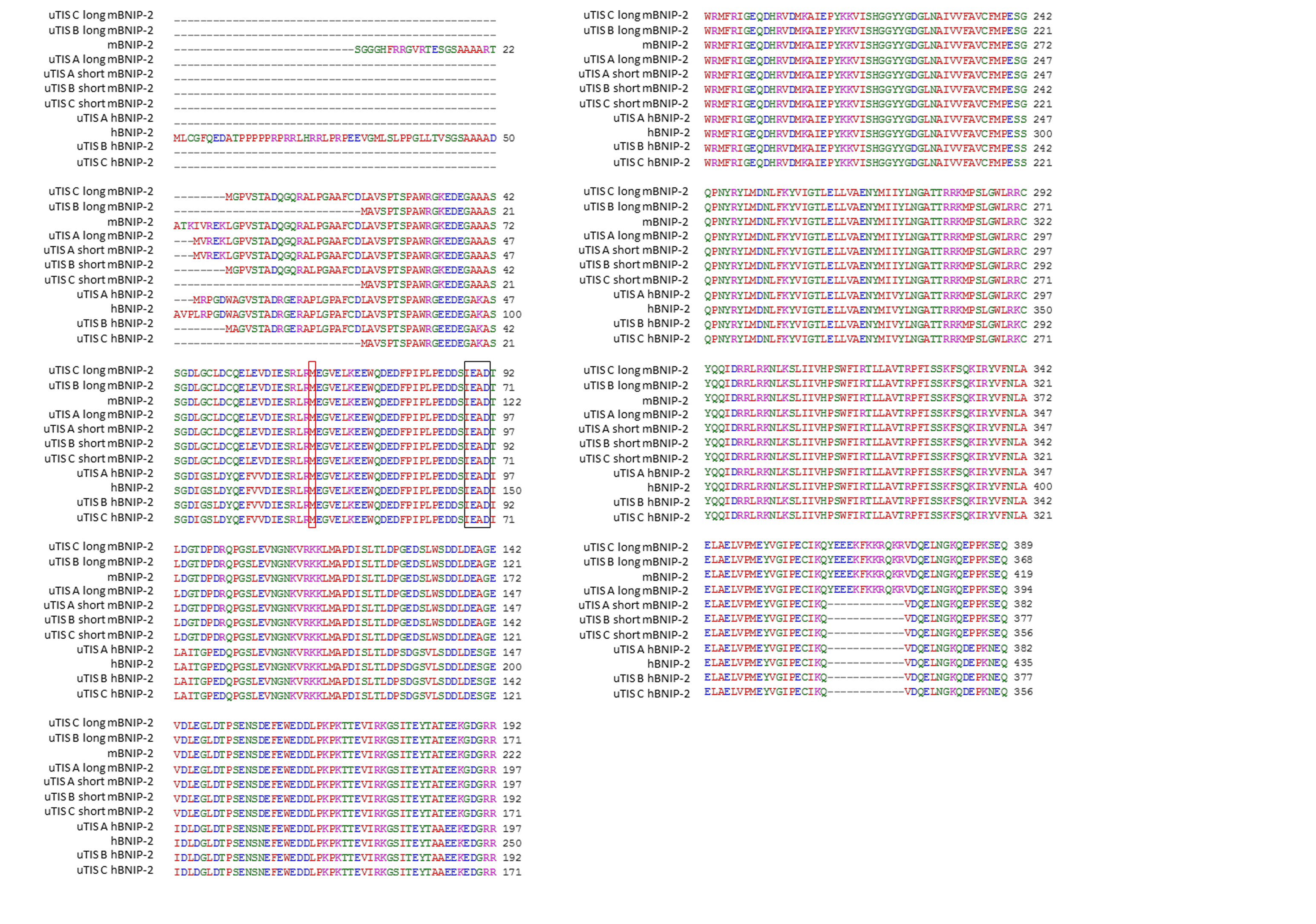

Supplement: Additional file 5: Figure S3 — ClustalW multiple sequence alignment of human and mouse BNIP-2 variants. The amino acid sequences of the longest hBNIP-2 annotated protein (annotated as hBNIP-2; UniProtKB accession [Trembl: J3KN59]) as well as the N-terminally extended uTIS variants of hBNIP2 [Swiss-Prot: Q12982] were used in the alignment. The longest mBNIP-2 variant is based on O54940 (Swiss-Prot) complemented with the 5′ leader from ENSMUSG00000011958 whereas other variants of mBNIP-2 (short mouse BNIP-2 [Trembl: Q91VL0] and long mBNIP-2 [Swiss-Prot: O54940]) are depicted together with their postulated N-terminal extensions. The black box highlights the P4-P1 IEAD recognition motif whereas the red box assigns the database annotated initiator Met of mouse and human BNIP-2 proteins. [file 1471-2091-15-21-S5.tiff]

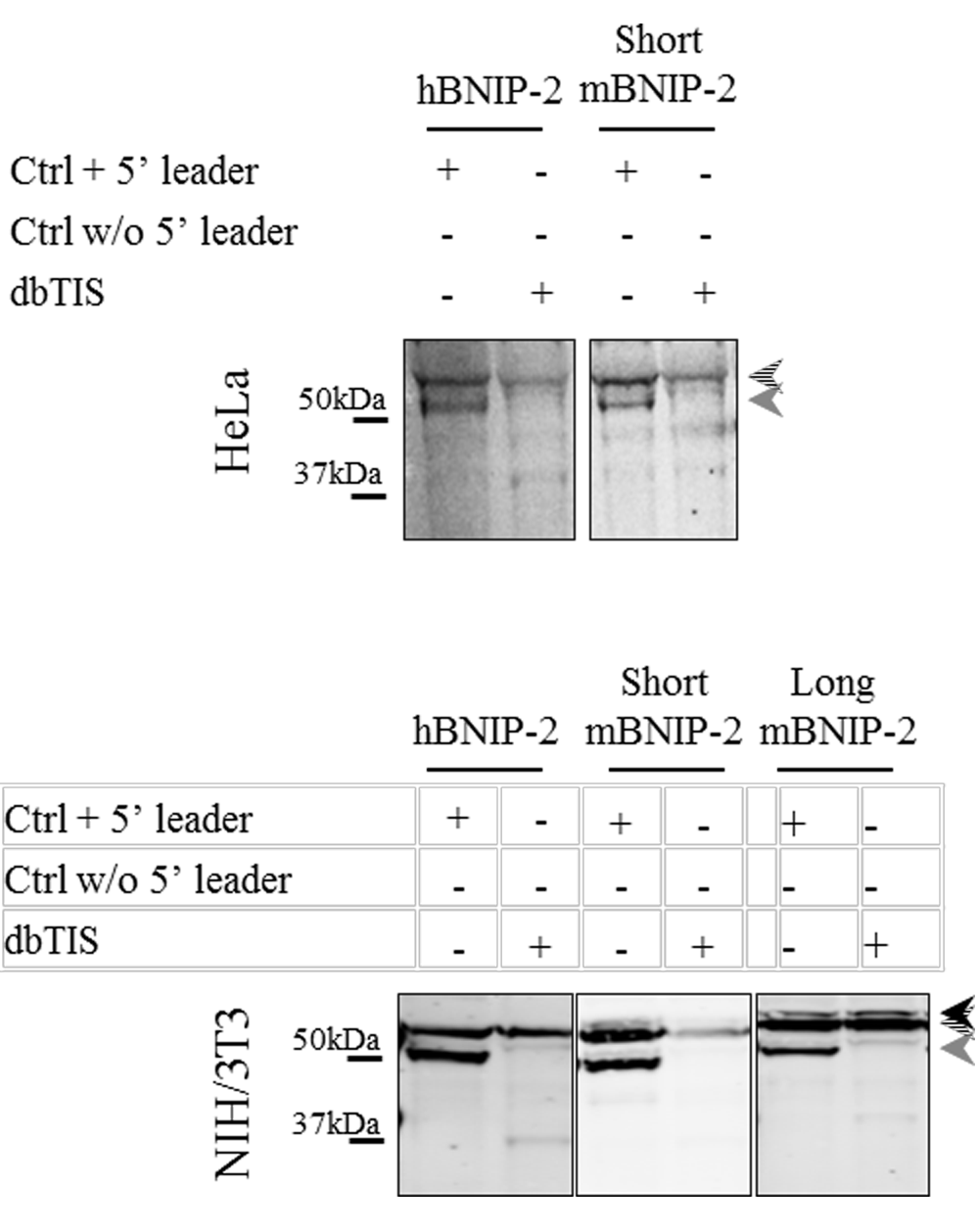

Supplement: Additional file 7: Figure S5 — Assessing the influence of 5′ leader sequences on BNIP-2 precursor patterns. Assignment of the precursor band corresponding to the database annotated protein (human BNIP-2:[Swiss-Prot: Q12982], short mouse BNIP-2:[Trembl: Q91VL0] and long mouse BNIP-2:[Swiss-Prot: O54940]) by mutating their respective initiator methionines. A grey arrow points to the UniProtKB annotated BNIP-2 variants, while black and dashed arrows indicate the BNIP-2 variants associated with translation initiation at uTIS B and uTIS C respectively. [file 1471-2091-15-21-S7.tiff]
