# Supplementary material for: Importance of extended protease substrate recognition motifs in steering BNIP-2 cleavage by human and mouse granzymes B
Source: BMC Biochem. 2014 Sep 10;15:21. doi: 10.1186/1471-2091-15-21 (PMC4169252; doi:10.1186/1471-2091-15-21)
Supplement: Additional file 6: Figure S4 — Cloned sequences of human (A), short mouse (B) and long mouse (C) BNIP-2. Alternative translation initiation sites are indicated in italics. Red and green letters denote the putative upstream translation initiation sites identified by means of ribosome profiling in mouse and human cells respectively. Below each nucleotide sequence, amino acid sequences of putative translation products are given. [file 1471-2091-15-21-S6.docx]

**Supplementary Figure S4**

1. Human BNIP-2

1 ctgtgtccgggtcagctgctgccgccgacgccgtaccg***ctg***cggccgggggat***tgg***gccg 60

61 gggtctccaccgccgaccgaggggagcgggctccgctcggccctgctttttgcgac***ctg***g 120

121 ccgtcagccccacgtcgccggcctggaggggcgaagaggacgagggggccaaggcttcct 180

181 ccggggacattggctccctggattatcaagagtttgtagttgacattgaatccaggctga 240

241 ggatggaaggtgtggaacttaaagaagaatggcaagatgaagattttccgatacctttac 300

301 cagaagatgatagtattgaagcagatatactagctataactggaccagaggaccagcctg 360

361 gctcactagaagttaatggaaataaagtgagaaagaaactaatggctccagacattagcc 420

421 tgacactggatcctagtgatggctctgtattgtcagatgatttggatgaaagtggggaga 480

481 ttgacttagatggcttagacacaccgtcagagaatagtaatgagtttgagtgggaagatg 540

541 atcttccaaaacccaagactactgaagtaattaggaaaggctcaattactgaatacacag 600

601 cagcagaggaaaaagaagatggacgacgctggcgtatgttcaggattggagaacaggacc 660

661 acagggttgatatgaaggcaattgaaccctataaaaaagttatcagccatgggggatatt 720

721 atggggatggattaaatgccattgttgtgtttgctgtctgtttcatgcctgaaagtagtc 780

781 agcctaactatagatacctgatggacaatctttttaaatatgttattggcactttggagc 840

841 tattagtagcagaaaactacatgatagtttatttaaatggtgcaacaactcgaagaaaaa 900

901 tgcccagtctgggatggctcaggaaatgttatcagcaaattgatagaaggttacggaaaa 960

961 atctaaaatccctaatcattgtacatccttcttggtttatcagaacacttctggctgtta 1020

1021 caagaccatttattagctcgaaattcagccaaaaaattagatacgtgtttaatttggcag 1080

1081 aactagcagaacttgtccccatggaatacgttggcataccagaatgcataaaacaagttg 1140

1141 atcaagaacttaatggaaaacaagatgaaccgaaaaatgaacag 1184

Protein sequence upon translation at uTIS A *(ctg)*:

**MRPGDWAGVSTADRGERAPLGPAFCDLAVSPTSPAWRGEEDEGAKASSGDIGSLDYQEFVVDIESRLR**MEGVELKEEWQDEDFPIPLPEDDSIEADILAITGPEDQPGSLEVNGNKVRKKLMAPDISLTLDPSDGSVLSDDLDESGEIDLDGLDTPSENSNEFEWEDDLPKPKTTEVIRKGSITEYTAAEEKEDGRRWRMFRIGEQDHRVDMKAIEPYKKVISHGGYYGDGLNAIVVFAVCFMPESSQPNYRYLMDNLFKYVIGTLELLVAENYMIVYLNGATTRRKMPSLGWLRKCYQQIDRRLRKNLKSLIIVHPSWFIRTLLAVTRPFISSKFSQKIRYVFNLAELAELVPMEYVGIPECIKQVDQELNGKQDEPKNEQ

Protein sequence upon translation at uTIS B *(tgg)*:

**MAGVSTADRGERAPLGPAFCDLAVSPTSPAWRGEEDEGAKASSGDIGSLDYQEFVVDIESRLR**MEGVELKEEWQDEDFPIPLPEDDSIEADILAITGPEDQPGSLEVNGNKVRKKLMAPDISLTLDPSDGSVLSDDLDESGEIDLDGLDTPSENSNEFEWEDDLPKPKTTEVIRKGSITEYTAAEEKEDGRRWRMFRIGEQDHRVDMKAIEPYKKVISHGGYYGDGLNAIVVFAVCFMPESSQPNYRYLMDNLFKYVIGTLELLVAENYMIVYLNGATTRRKMPSLGWLRKCYQQIDRRLRKNLKSLIIVHPSWFIRTLLAVTRPFISSKFSQKIRYVFNLAELAELVPMEYVGIPECIKQVDQELNGKQDEPKNEQ

Protein sequence upon translation at uTIS C *(ctg)*:

**MAVSPTSPAWRGEEDEGAKASSGDIGSLDYQEFVVDIESRLR**MEGVELKEEWQDEDFPIPLPEDDSIEADILAITGPEDQPGSLEVNGNKVRKKLMAPDISLTLDPSDGSVLSDDLDESGEIDLDGLDTPSENSNEFEWEDDLPKPKTTEVIRKGSITEYTAAEEKEDGRRWRMFRIGEQDHRVDMKAIEPYKKVISHGGYYGDGLNAIVVFAVCFMPESSQPNYRYLMDNLFKYVIGTLELLVAENYMIVYLNGATTRRKMPSLGWLRKCYQQIDRRLRKNLKSLIIVHPSWFIRTLLAVTRPFISSKFSQKIRYVFNLAELAELVPMEYVGIPECIKQVDQELNGKQDEPKNEQ

1. Short mouse BNIP-2

1 ccacgcgtccgtgcagccgcccggactgcaactaag***atc***gtccgggaaaag***ctg***ggcccc 60

61 gtctccacagccgaccaagggcagcgggctctgcccggcgccgctttctgcgac***ctg***gcc 120

121 gtcagccccacgtcgccggcctggaggggcaaagaggacgagggggccgcggcttcctcc 180

181 ggggaccttggctgcctggattgccaggagctggaagttgacattgagtctaggctgagg 240

241 atggaaggtgtggagctgaaggaagaatggcaggatgaagattttccaatacctttacca 300

301 gaagatgacagcattgaagcagatacactagatggaactgatccagacagacagcctggc 360

361 tccttagaagttaatgggaacaaagtaaggaagaaactgatggccccagacatcagcctg 420

421 accctggatcctggtgaagactctctgtggtccgatgatttggatgaagctggagaggtt 480

481 gacctggaaggcttagacaccccatcagagaacagtgatgagtttgagtgggaagatgat 540

541 cttcccaaacccaaaactactgaagtcattaggaaaggctcgattactgagtacacagcc 600

601 acagaagaaaagggcgatggacgccgctggcgcatgttcaggattggagagcaggaccac 660

661 agggtggacatgaaggcgatcgagccctacaaaaaagttattagccatggaggatattat 720

721 ggggacggcttaaatgccattgttgtatttgctgtctgtttcatgcctgagagtggtcag 780

781 cctaactatagatatttgatggacaatctctttaaatatgttattggcactttagagctg 840

841 ttggtagcagagaactacatgatcatttacttaaatggtgcgacaactcgaagaaaaatg 900

901 cccagtttgggatggctcaggagatgctaccagcaaattgatagacgattaaggaaaaac 960

961 cttaagtctctaatcattgtgcacccctcttggtttatccgaacacttctggctgttaca 1020

1021 cgaccatttatcagttcaaagttcagtcagaagatcagatacgtctttaacttggccgag 1080

1081 ctggcagagcttgttcccatggagtatgtcggcataccagagtgcatcaaacaagttgat 1140

1141 caagagcttaatggaaaacaagaaccaccaaaaagtgagcagtaa 1185

Protein sequence upon translation at uTIS A *(atc)*:

**MVREKLGPVSTADQGQRALPGAAFCDLAVSPTSPAWRGKEDEGAAASSGDLGCLDCQELEVDIESRLR**MEGVELKEEWQDEDFPIPLPEDDSIEADTLDGTDPDRQPGSLEVNGNKVRKKLMAPDISLTLDPGEDSLWSDDLDEAGEVDLEGLDTPSENSDEFEWEDDLPKPKTTEVIRKGSITEYTATEEKGDGRRWRMFRIGEQDHRVDMKAIEPYKKVISHGGYYGDGLNAIVVFAVCFMPESGQPNYRYLMDNLFKYVIGTLELLVAENYMIIYLNGATTRRKMPSLGWLRRCYQQIDRRLRKNLKSLIIVHPSWFIRTLLAVTRPFISSKFSQKIRYVFNLAELAELVPMEYVGIPECIKQVDQELNGKQEPPKSEQ

Protein sequence upon translation at uTIS B *(ctg)*:

**MGPVSTADQGQRALPGAAFCDLAVSPTSPAWRGKEDEGAAASSGDLGCLDCQELEVDIESRLR**MEGVELKEEWQDEDFPIPLPEDDSIEADTLDGTDPDRQPGSLEVNGNKVRKKLMAPDISLTLDPGEDSLWSDDLDEAGEVDLEGLDTPSENSDEFEWEDDLPKPKTTEVIRKGSITEYTATEEKGDGRRWRMFRIGEQDHRVDMKAIEPYKKVISHGGYYGDGLNAIVVFAVCFMPESGQPNYRYLMDNLFKYVIGTLELLVAENYMIIYLNGATTRRKMPSLGWLRRCYQQIDRRLRKNLKSLIIVHPSWFIRTLLAVTRPFISSKFSQKIRYVFNLAELAELVPMEYVGIPECIKQVDQELNGKQEPPKSEQ

Protein sequence upon translation at uTIS C *(ctg)*:

**MAVSPTSPAWRGKEDEGAAASSGDLGCLDCQELEVDIESRLR**MEGVELKEEWQDEDFPIPLPEDDSIEADTLDGTDPDRQPGSLEVNGNKVRKKLMAPDISLTLDPGEDSLWSDDLDEAGEVDLEGLDTPSENSDEFEWEDDLPKPKTTEVIRKGSITEYTATEEKGDGRRWRMFRIGEQDHRVDMKAIEPYKKVISHGGYYGDGLNAIVVFAVCFMPESGQPNYRYLMDNLFKYVIGTLELLVAENYMIIYLNGATTRRKMPSLGWLRRCYQQIDRRLRKNLKSLIIVHPSWFIRTLLAVTRPFISSKFSQKIRYVFNLAELAELVPMEYVGIPECIKQVDQELNGKQEPPKSEQ

1. Long mouse BNIP-2

1 gactgaatccgggtcagctgcagccgcccggactgcaactaag***atc***gtccgggaaaag***ct*** 60

61 ***g***ggccccgtctccacagccgaccaagggcagcgggctctgcccggcgccgctttctgcga 120

121 c***ctg***gccgtcagccccacgtcgccggcctggaggggcaaagaggacgagggggccgcggc 180

181 ttcctccggggaccttggctgcctggattgccaggagctggaagttgacattgagtctag 240

241 gctgaggatggaaggtgtggagctgaaggaagaatggcaggatgaagattttccaatacc 300

301 tttaccagaagatgacagcattgaagcagatacactagatggaactgatccagacagaca 360

361 gcctggctccttagaagttaatgggaacaaagtaaggaagaaactgatggccccagacat 420

421 cagcctgaccctggatcctggtgaagactctctgtggtccgatgatttggatgaagctgg 480

481 agaggttgacctggaaggcttagacaccccatcagagaacagtgatgagtttgagtggga 540

541 agatgatcttcccaaacccaaaactactgaagtcattaggaaaggctcgattactgagta 600

601 cacagccacagaagaaaagggcgatggacgccgctggcgcatgttcaggattggagagca 660

661 ggaccacagggtggacatgaaggcgatcgagccctacaaaaaagttattagccatggagg 720

721 atattatggggacggcttaaatgccattgttgtatttgctgtctgtttcatgcctgagag 780

781 tggtcagcctaactatagatatttgatggacaatctctttaaatatgttattggcacttt 840

841 agagctgttggtagcagagaactacatgatcatttacttaaatggtgcgacaactcgaag 900

901 aaaaatgcccagtttgggatggctcaggagatgctaccagcaaattgatagacgattaag 960

961 gaaaaaccttaagtctctaatcattgtgcacccctcttggtttatccgaacacttctggc 1020

1021 tgttacacgaccatttatcagttcaaagttcagtcagaagatcagatacgtctttaactt 1080

1081 ggccgagctggcagagcttgttcccatggagtatgtcggcataccagagtgcatcaaaca 1140

1141 gtatgaagaagaaaagtttaaaaagagacaaaaaagagttgatcaagagcttaatggaaa 1200

1201 acaagaaccaccaaaaagtgagcagtaa 1228

Protein sequence upon translation at uTIS A *(atc)*:

**MVREKLGPVSTADQGQRALPGAAFCDLAVSPTSPAWRGKEDEGAAASSGDLGCLDCQELEVDIESRLR**MEGVELKEEWQDEDFPIPLPEDDSIEADTLDGTDPDRQPGSLEVNGNKVRKKLMAPDISLTLDPGEDSLWSDDLDEAGEVDLEGLDTPSENSDEFEWEDDLPKPKTTEVIRKGSITEYTATEEKGDGRRWRMFRIGEQDHRVDMKAIEPYKKVISHGGYYGDGLNAIVVFAVCFMPESGQPNYRYLMDNLFKYVIGTLELLVAENYMIIYLNGATTRRKMPSLGWLRRCYQQIDRRLRKNLKSLIIVHPSWFIRTLLAVTRPFISSKFSQKIRYVFNLAELAELVPMEYVGIPECIKQYEEEKFKKRQKRVDQELNGKQEPPKSEQ

Protein sequence upon translation at uTIS B *(ctg)*:

**MGPVSTADQGQRALPGAAFCDLAVSPTSPAWRGKEDEGAAASSGDLGCLDCQELEVDIESRLR**MEGVELKEEWQDEDFPIPLPEDDSIEADTLDGTDPDRQPGSLEVNGNKVRKKLMAPDISLTLDPGEDSLWSDDLDEAGEVDLEGLDTPSENSDEFEWEDDLPKPKTTEVIRKGSITEYTATEEKGDGRRWRMFRIGEQDHRVDMKAIEPYKKVISHGGYYGDGLNAIVVFAVCFMPESGQPNYRYLMDNLFKYVIGTLELLVAENYMIIYLNGATTRRKMPSLGWLRRCYQQIDRRLRKNLKSLIIVHPSWFIRTLLAVTRPFISSKFSQKIRYVFNLAELAELVPMEYVGIPECIKQYEEEKFKKRQKRVDQELNGKQEPPKSEQ

Protein sequence upon translation at uTIS C *(ctg)*:

**MAVSPTSPAWRGKEDEGAAASSGDLGCLDCQELEVDIESRLR**MEGVELKEEWQDEDFPIPLPEDDSIEADTLDGTDPDRQPGSLEVNGNKVRKKLMAPDISLTLDPGEDSLWSDDLDEAGEVDLEGLDTPSENSDEFEWEDDLPKPKTTEVIRKGSITEYTATEEKGDGRRWRMFRIGEQDHRVDMKAIEPYKKVISHGGYYGDGLNAIVVFAVCFMPESGQPNYRYLMDNLFKYVIGTLELLVAENYMIIYLNGATTRRKMPSLGWLRRCYQQIDRRLRKNLKSLIIVHPSWFIRTLLAVTRPFISSKFSQKIRYVFNLAELAELVPMEYVGIPECIKQYEEEKFKKRQKRVDQELNGKQEPPKSEQ
